# Functional Diversification of *Populus* FLOWERING LOCUS D-LIKE3 Transcription Factor and Two Paralogs in Shoot Ontogeny, Flowering, and Vegetative Phenology

**DOI:** 10.3389/fpls.2022.805101

**Published:** 2022-02-03

**Authors:** Xiaoyan Sheng, Chuan-Yu Hsu, Cathleen Ma, Amy M. Brunner

**Affiliations:** ^1^Department of Forest Resources and Environmental Conservation, Virginia Tech, Blacksburg, VA, United States; ^2^Institute for Genomics, Biocomputing and Biotechnology, Mississippi State University, Starkville, MS, United States; ^3^Department of Forest Ecosystems and Society, Oregon State University, Corvallis, OR, United States

**Keywords:** *FLOWERING LOCUS D*, *FT*, *FRUITFULL*, gene duplication, heterochrony, leaf development, phenology, secondary growth

## Abstract

Both the evolution of tree taxa and whole-genome duplication (WGD) have occurred many times during angiosperm evolution. Transcription factors are preferentially retained following WGD suggesting that functional divergence of duplicates could contribute to traits distinctive to the tree growth habit. We used gain- and loss-of-function transgenics, photoperiod treatments, and circannual expression studies in adult trees to study the diversification of three *Populus FLOWERING LOCUS D-LIKE (FDL)* genes encoding bZIP transcription factors. Expression patterns and transgenic studies indicate that *FDL2.2* promotes flowering and that *FDL1* and *FDL3* function in different vegetative phenophases. Study of dominant repressor *FDL* versions indicates that the FDL proteins are partially equivalent in their ability to alter shoot growth. Like its paralogs, *FDL3* overexpression delays short day-induced growth cessation, but also induces distinct heterochronic shifts in shoot development—more rapid phytomer initiation and coordinated delay in both leaf expansion and the transition to secondary growth in long days, but not in short days. Our results indicate that both regulatory and protein coding sequence variation contributed to diversification of *FDL* paralogs that has led to a degree of specialization in multiple developmental processes important for trees and their local adaptation.

## Introduction

Distinguishing features of trees include large crowns enabled by extensive wood development and protracted flowering-incompetent phases. Central to woody shoot development is the transition from primary growth—the production of phytomers and stem elongation initiated by the shoot apical meristem (SAM) and rib meristem—to secondary growth, which commences with the formation of a vascular cambium that increases girth by producing secondary xylem (Spicer and Groover, 2010; McKim, 2019). In *Populus*, this transition is synchronized with leaf maturation (Larson and Isebrands, 1971, 1974). Trees inhabit and often dominate temperate and boreal regions because they evolved the ability to become endodormant and orchestrate cellular adaptations that enable above ground meristems and tissues to survive winter freezing temperatures and dehydration stress (Howe et al., 2003; Preston and Sandve, 2013). The tree growth habit has been lost and gained many times throughout angiosperm evolution (Groover, 2005), which has been characterized by whole-genome duplications (WGDs) as well as segmental and tandem duplications (Hanada et al., 2008; Soltis et al., 2015). Although these events are likely to have had a major role in the repeated evolution of trees, empirical evidence for the role of gene duplicate diversification in processes that define the tree life style is sparse.

Flowering time in diverse plants is cued by indicators of seasonal change, with photoperiod and an extended period of chilling temperatures typically major signals (Bernier and Perilleux, 2005). Vegetative phenology of trees is also cued by these signals and study of tree homologs of Arabidopsis flowering time genes provided some of the first evidence for the contribution of gene duplicate diversification to tree developmental processes (reviewed in Ding and Nilsson, 2016; Brunner et al., 2017). Although reproductive phenology is integrated with vegetative phenology in adult trees, their phenophases are not always coincident or controlled by the same environmental cue. Long days (LDs) induce expression of the transcriptional co-factor and florigen *FLOWERING LOCUS* (*FT*) in Arabidopsis leaves and *FT* homologs in diverse plants have conserved functions in the floral transition (Abe et al., 2005; Wigge et al., 2005; Corbesier et al., 2007; Taoka et al., 2011). In *Populus*, *FT2* is expressed in leaves and rapidly downregulated by short days (SDs), whereas *FT1* expression peaks during winter in multiple tissues within winter buds (Böhlenius et al., 2006; Hsu et al., 2011). Changes in *cis*-regulatory sequences have generally been considered the predominant mechanism for developmental evolution, but increasing evidence supports a role for protein coding changes and both types of sequence changes can be necessary for the evolution of new transcriptional circuits (Lynch and Wagner, 2008; Bartlett, 2020; Britton et al., 2020). Although their divergent seasonal expression patterns could be sufficient for functional diversification of the *Populus FT* paralogs, their encoded proteins are not fully equivalent. FT1 is much more effective than FT2 at inducing flowering, suggesting that FT1 could mediate the transition of incipient axillary meristems to inflorescence meristems within winter buds and also promote endodormancy release (Hsu et al., 2011; Rinne et al., 2011; Brunner et al., 2014). Conversely, overexpression of either paralog delayed SD-induced growth cessation, but only *FT2* expression is consistent with a growth-promoting function (Böhlenius et al., 2006; Hsu et al., 2011).

A conserved mechanism to promote flowering centers on a complex involving FT and the bZIP transcription factor FLOWERING LOCUS D (FD) that activates the related MADS-box genes *FRUITFULL (FUL)* in the SAM and *APETALA1 (AP1)* in lateral floral meristems (Schmid et al., 2003; Abe et al., 2005; Wigge et al., 2005; Torti et al., 2012). The FT-FD module has additional effects under certain environmental conditions and different FT and FD homologs appear to have roles in other developmental processes. For example, in SDs, *35S::FT* Arabidopsis transgenics have small, curled leaves and this phenotype is dependent on *FD* (Teper-Bamnolker and Samach, 2005). Both rice OsFD1 and OsFD2 can form a complex with FT homologs; however, only *OsFD1* promoted flowering whereas *OsFD2* overexpression affected shoot branching and panicle architecture (Taoka et al., 2011; Tsuji et al., 2013; Brambilla et al., 2017).

Transcription factors are preferentially retained after WGDs, which also provide opportunity for a duplicate regulatory module to evolve in concert (Maere et al., 2005; Freeling, 2009; Wu et al., 2020). The *Populus* genome contains three *FD-LIKE* (*FDL*) genes (Supplementary Figure S1). As is the case for *FT1* and *FT2*, *FDL1* and *FDL2* resulted from the Salicoid WGD, estimated to have occurred ~60 Ma (Tuskan et al., 2006; Rodgers-Melnick et al., 2012). The growth and morphology of *35S::FDL1* poplar transgenics were similar to wild-type (WT) under LDs, but bud set was delayed under SDs (Tylewicz et al., 2015). FDL1 interacts with ABSCISIC ACID-INSENSITIVE3 (ABI3), a bZIP transcription factor with a role in bud formation (Rohde, 2002; Ruttink et al., 2007; Tylewicz et al., 2015). Overexpression of *ABI3* or *FDL1* upregulates some of the same genes linked to bud development and stress adaptation, suggesting a role for *FDL1* in these processes. Two splice variants of *FDL2* induced different phenotypes. Transgenics overexpressing *FDL2.1* were dwarf, but their SD growth response did not differ from WT (Tylewicz et al., 2015). Under LDs, *35S::FDL2.2* (referred to as *FD1* in Parmentier-Line and Coleman, 2016), poplar transgenics flowered precociously and had small leaves and increased branching, but similar to *FDL1* overexpression, SD-induced bud set was delayed.

Here, we report that both regulatory and protein coding divergence contribute to the varying degrees of functional diversification among the three *FDL* genes. Adding new information to previous studies (Tylewicz et al., 2015; Parmentier-Line and Coleman, 2016), we show that only *FDL2.2* can induce precocious flowering and its strong upregulation in developing spring inflorescence buds supports a primary role in the floral transition. Furthermore, we show that the vegetative expression of *FDL1* and *FDL3* peak at opposite seasons, suggesting diversified roles in phenology. *FDL3* overexpression showed a novel photoperiod-dependent phenotype. In LDs, *FDL3* induced a delay in leaf maturation and the transition to secondary growth and altered the expression of developmentally responsive gibberellin (GA) synthesis and response genes. Expression studies of *AP1/FUL* homologs suggest that duplicate FT-FD-AP1/FUL modules could play distinct roles in vegetative and reproductive development.

## Materials and Methods

### Binary Constructs and Plant Transformation

The *Populus deltoides FDL2.2* and *FDL3* coding regions were amplified with *Pfu* DNA polymerase (Stratagene) and inserted into pGEM-T Easy vector (Promega). For dominant repression constructs, the 3′ end of coding regions were extended to encode the SRDX repressor domain (rd; Hiratsu et al., 2003) by designing a reverse primer containing the SRDX coding sequence (LDLDLELRLGFS). All primer sequences are provided in Supplementary Table S1. The coding sequences were excised by BamHI/KpnI digestion and cloned into the pBI121 binary vector (BD Biosciences). Vectors were introduced into *Agrobacterium tumefaciens* strain GV3101 and transformed into *Populus tremula* × *Populus alba* clone INRA 717-1B4, hereafter referred to as wild type (WT), as previously described (Meilan and Ma, 2006).

### Plant Growth Conditions and Measurements

All transgenic and non-transgenic WT plants were propagated *in vitro*. Rooted plantlets were transferred from tissue culture to soil (Promix B, Canada) and acclimated in a growth chamber. After acclimation, plants were transferred to two-gallon pots and provided with 48 g Osmocote Plus 15-9-12 fertilizer/pot approximately 3 weeks after transfer. LD growth chamber conditions were 16-h light/8-h dark, with light intensity of 100 μ mol m^_2^ s^_1^ at plant level, 20°C–22°C, and 65% relative humidity. For SD conditions, the photoperiod was reduced to 8 h by changing the end of day time. In the greenhouse, ambient daylength was extended to 16 h using high pressure sodium lamps. Leaf plastochron index (LPI) was adopted for measurements and collecting samples (Larson and Isebrands, 1971). LPI1 was defined as the first leaf below the shoot apex (SA) with a lamina length of at least 1 cm. The internode (IN) directly beneath the LPI1 leaf was designated as IN1.

### Gene Expression

The *P. deltoides* samples for seasonal gene expression studies and parameters for qRT-PCR were the same as previously described (Hsu et al., 2011), and sampling is summarized in Supplementary Table S4. To study expression in different tissues, we sampled 4-month-old WT plants grown in LD greenhouse conditions. For SA samples, all leaves visible to the naked eye were removed. Axillary buds (ABs) were collected from LPI10 to LPI20. Young leaf (YL) was from LPI2, and nearly mature leaf (ML) was LPI6 (leaf length ~75% of fully expanded leaf size). IN2 is in the primary growth zone, whereas IN6 is transitioning from primary to secondary growth. Phloem (Ph) and xylem (Xy) were scraped from the stem undergoing secondary growth below IN6. We collected non-woody lateral roots. All samples were collected 2 h after the start of the light period. All samples were immediately frozen in liquid nitrogen and stored at −80°C. Samples from three or more trees were pooled for RNA extraction using the RNeasy Plant Mini Kit (Qiagen) and RNase-Free DNase Set (Qiagen) as previously described (Brunner et al., 2004). Each cDNA was synthesized from 2.0 μg total RNA and an oligo (dT) primer using the High Capacity cDNA reverse transcription kit (Applied Biosystems), according to the manufacturer’s protocol. We used the Power SYBR Green PCR Master Mix kit (Applied Biosystems) and the ABI PRISM™ 7500 Real-Time PCR system (Applied Biosystems) for qRT-PCR reactions with three replications per RNA sample. The PCR program was set up to perform an initial incubation at 95°C for 10 min, followed by 95°C for 15 s, and 60°C for 1 min, for a total of 40 cycles. To enable design of gene-specific primers, the more divergent 3′ coding and untranslated region (UTR) were isolated for three aspen *AP1/FUL* family members using 3′ Rapid Amplification of cDNA Ends (3′ RACE). For *LAP1a* and *LAP1b*, full-length aspen cDNA sequences were already available (GenBank accession numbers AF034093 and AF034094). Using *P. tremula × P. alba* 717-1B4 cDNA as template, 3′ regions were amplified using gene-specific primers and a 3′ RACE adapter primer. The resulting DNA fragment was ligated into a shuttle vector, pCR 2.1 (TA Cloning Kit, Invitrogen, Carlsbad, CA, United States), and sequenced. 3′ RACE sequences and alignment of 3′ regions of *AP1/FUL* sequences from different *Populus* species/hybrids and location of primers are shown in Supplementary Figure S2. All primers are listed in Supplementary Table S1. We used an ubiquitin gene (*UBQ2*) as an internal reference (Mohamed et al., 2010) and normalized the Ct values across plates, determining relative quantities using comparative Ct method (2^−ΔΔCt^) as previously described (Livak and Schmittgen, 2001).

For *in situ* hybridization, immature inflorescences were collected from wild *Populus trichocarpa* trees near Corvallis, OR, United States, and fixed and embedded as previously described (Kelly et al., 1995). Transcripts were detected using antisense riboprobes from the 3′ ends (Supplementary Figure S2) of *P. trichocarpa LAP1a* (396 bp) and *LAP1b* (360 bp) cDNAs. Sequences were cloned into pBluscript KS and antisense and sense digoxygenin (DIG) labeled transcripts were produced with T3 and T7 RNA polymerases and DIG RNA Labeling Kit (Roche). Hybridization was done with DIG-labeled T3 and T7 probes (0.5 ng/μl) at 45°C overnight. Hybridized probes were detected by application of Anti-Dig Fab conjugated with alkaline phosphatase (1:1,250 dilution, Roche) and nitroblue tetrazolium/5-bromo-4-chloro-3-indoyl-phosphate.

### Microscopic Analysis

For analysis of primary growth and transitional growth internodes, samples were immobilized in 5% agarose and sectioned (60 μm thickness) with a vibratome (Leica VT1200). We sectioned secondary growth internodes with a GSL1-microtome (Swiss Federal Institute for Forest, Snow and Landscape Research WSL, Switzerland). Sections were stained in a drop of the following solution: 1 g phloroglucinol (Sigma-Aldrich) in 100 ml, 95% EtOH, and 16 ml 37% HCL. Procedures for embedding and histology of stem samples and *in situ* hybridization of developing inflorescences are provided in the section “Materials and Methods.” All images were captured using a Zeiss Axio Imager A1 (Carl Zeiss, Oberkochen, Germany). For more detailed images of stem anatomy (Supplementary Figure S6), samples were fixed and embedded in LR white resin (London Resin Company, Ltd.) as previously described (Grant et al., 2010) and sectioned (2 μm thickness) with glass knives (Leica RM2265). Sections were stained with Toluidine blue/boric acid (0.05% w/v) for 1 min.

### Phylogenetic Analysis

FLOWERING LOCUS D family protein sequences (Supplementary Table S2) were aligned using MUSCLE (Edgar, 2004). A maximum-likelihood phylogenetic analysis was performed on the sequence alignment using the JTT + G model, a site coverage cut-off of 90% for alignment gaps/missing data and 100 bootstraps for branch support testing with the program MEGA7 (Kumar et al., 2016). Analysis of AP1/FUL family members (Supplementary Table S3) was the same except that all positions with less than 50% site coverage were eliminated.

### Statistics Analyses

Height, leaf length, and new leaf formation data were analyzed in JMP Pro 12 (SAS Institute Inc., 2016–2017), using the Fit model to test the effects of constructs and, when applicable, the events within constructs. We used two-sample *t*-test to evaluate differences between transgenic and WT means and to test differences between multiple group means, we used the LSMEANS protocol and applied the Tukey–Kramer’s adjustment for all possible pairwise comparisons between group means.

## Results

### *FDL* Genes Differ in Reproductive and Seasonal Vegetative Expression

We cloned full-length cDNAs of the three *FDL* genes from *P. deltoides*. *FDL1* and the *FDL2.1* and *FDL2.2* splice variants encode proteins nearly identical to those previously reported from *P. trichocarpa* (Tylewicz et al., 2015; Parmentier-Line and Coleman, 2016) except that the FDL1 reported here contains an additional 39 amino acids at its N-terminus (Supplementary Figure S1). FDL3 shares the conserved C-terminal phosphorylation (T)/SAP motif (Tsuji et al., 2013) and groups with FD in a phylogenetic tree (Supplementary Figure S1).

**Figure 1:**
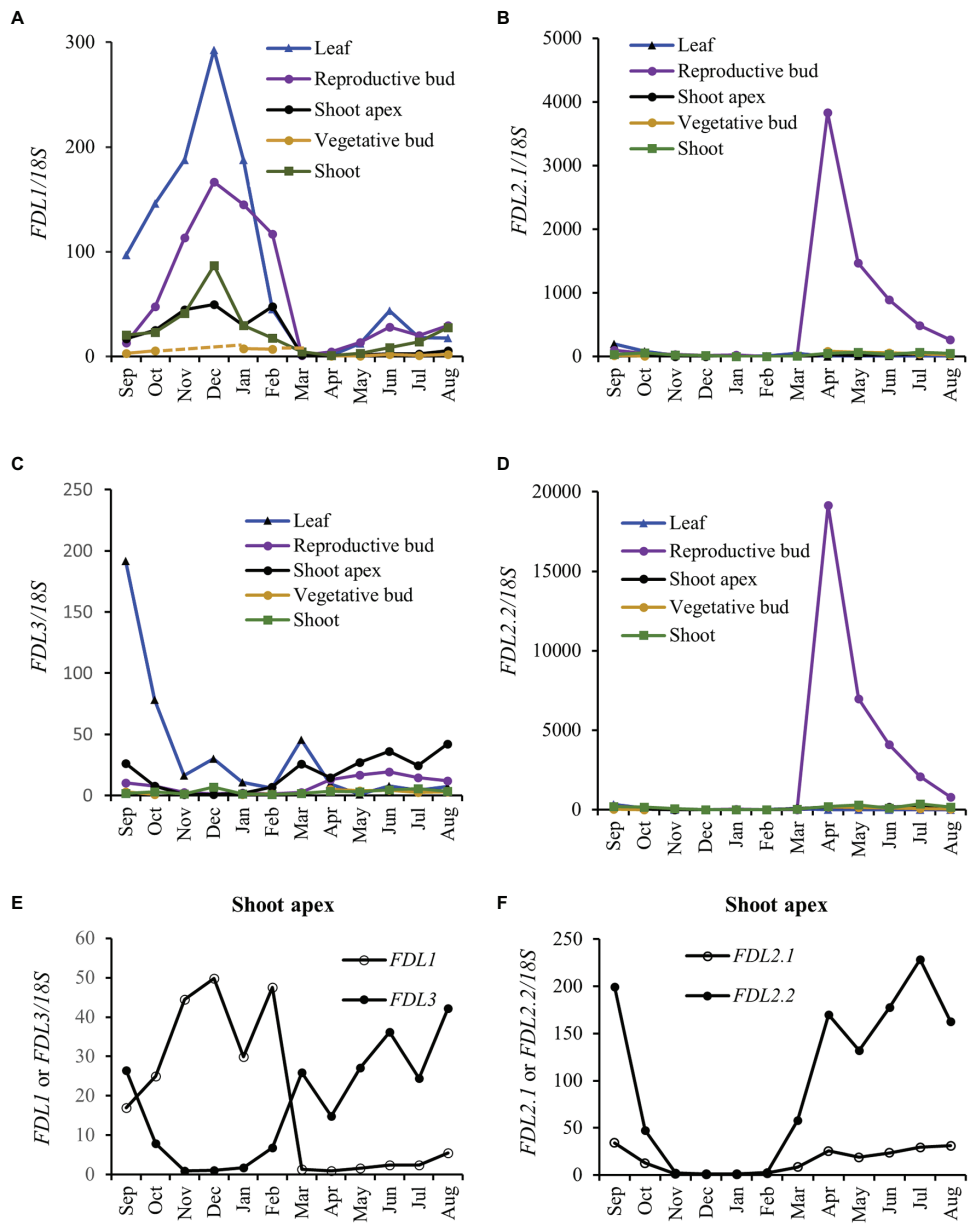
*FLOWERING LOCUS D-LIKE* (*FDL*) genes differ in regulation. Relative expression is fold change in transcript levels of **(A)** *FDL1*, **(B)** *FDL2.1*, **(C)** *FDL3*, and **(D)** *FDL2.2* relative to the time point with the lowest expression within a tissue (*n* = 3, except for shoot apex where the three apices were pooled to provide sufficient sample for analysis). *FDL* expression was normalized against reference gene *18S rRNA*. **(E)** *FDL1* and *FDL3* and **(F)** *FDL2.1* and *FDL2.2* expression in shoot apices is presented separately to allow comparison of circannual patterns among the different *FDLs*. Axillary reproductive bud flush began in late February with anthesis reached in March (April sample is newly initiated floral bud). From September to March, preformed leaves and shoots were dissected from terminal buds.

We studied *FDL* expression in different vegetative tissues and developmental stages of 4-month-old WT trees grown in a LD greenhouse. Both *FDL2* splice variants and *FDL3* showed highest expression in shoot apices, whereas *FDL1* showed highest expression in a transitional growth internode (Supplementary Figure S3). Study of circannual expression of *Populus FT* paralogs in different tissues was instrumental in revealing the divergence of *FT1* and *FT2* functions (Hsu et al., 2011). Hence, we studied expression of the *FDL*s using these same samples collected from adult *P. deltoides* growing in Mississippi, United States. Consistent with its ability to induce flowering (Parmentier-Line and Coleman, 2016), *FDL2.2* was highly upregulated in newly developing reproductive buds as was the *FDL2.1* splice variant (Figures 1B,D). The highest seasonal expression of *FDL1* was during late autumn–winter (Figure 1A), consistent with its indicated role in mediating bud maturation and cold adaptation (Tylewicz et al., 2015). *FDL3* was more highly expressed in shoot apices during the growing season compared to autumn–winter season and was transiently upregulated in leaves during early autumn at the timepoint when leaf collection shifted from fully expanded leaf (August) to preformed leaf within a terminal bud (September; Figures 1C,E). In shoot apices, the seasonal expression pattern of both *FDL3* and *FDL2* was opposite of *FDL1*’s expression pattern (Figures 1E,F).

### Dominant Repressor Versions of the Poplar *FDL* Genes Reduce Shoot Elongation to Different Degrees

**Figure 2:**
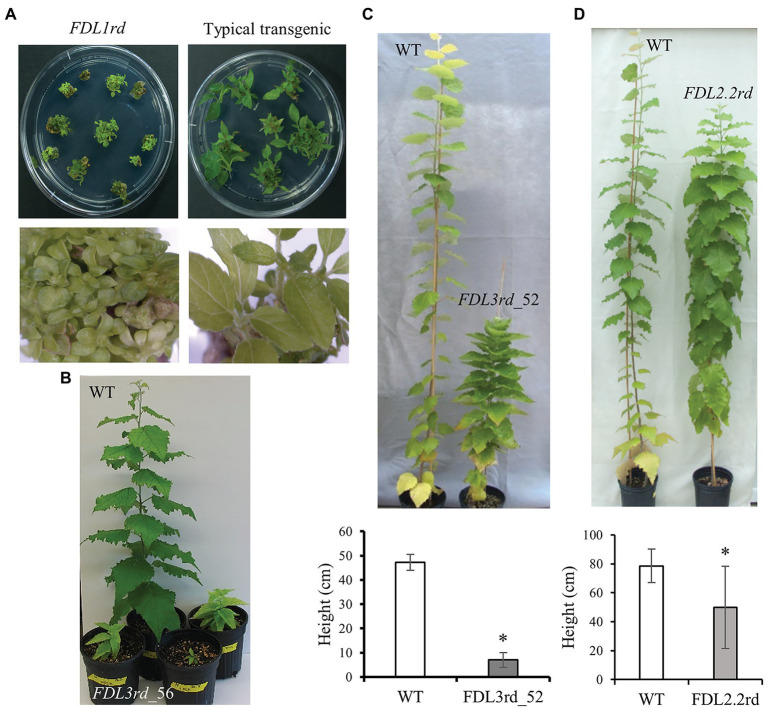
Phenotypic effects of dominant repressor versions of *FDL* genes. **(A)** *FDL1rd* transgenic shoots on shoot elongation medium compared to an unrelated transgenic regenerated at the same time that displays typical shoot elongation. Each clump of shoots corresponds to a single explant that was induced to form callus and then shoots. Bottom photos show shoots from one of the explants in the top photos. **(B)** *FDL3rd_56* transgenics showed reduced shoot growth and set terminal buds within 2 months after potting under LD conditions, whereas WT continued to grow. **(C)** *FDL3rd_52* trees showed reduced shoot elongation. Representative 6-month-old trees are shown and values are means ± SE for two WT and two *FDL3rd_52* trees after 3 months of growth in a greenhouse. **(D)** Representative 6-month-old WT and *FDL2.2rd* trees and mean heights ± SE after 4 months of growth in a greenhouse. For WT, *n* = 12; For *FDL2.2rd*, *n* = 16 (eight events with two ramets/event). ^*^p < 0.01 compared to WT.

To compare protein functional equivalency, we first extended each *FDL* coding sequence to encode the SRDX repressor domain (Hiratsu et al., 2003), and then, each sequence was placed under the control of the 35S promoter and nos terminator. The *FDL1rd* transgene imposed the most severe effect on shoot development (Figure 2A). Many tiny transgenic shoots, confirmed as positive for the *FDL1rd* transgene by PCR, regenerated from callus. However, *FDL1rd* transgenics failed to elongate when sub-cultured on shoot elongation medium and we could not regenerate any rooted plants. Six independent transgenic events of *FDL2.1rd* were rooted and four events showed short internodes whereas the other two showed WT-like growth *in vitro* (Supplementary Figure S4A). For transcriptional activators, overexpression of rd-modified and WT proteins is expected to induce opposite phenotypes; however, overexpression of *FDL2.1* also reduced shoot growth (Tylewicz et al., 2015). As this suggests that addition of the rd augmented WT FDL2.1 function rather than induced a loss-of-function phenotype, *FDL2.1rd* transgenics were not studied further. Eleven events of *FDL2.2rd* were rooted, but only five events showed reduced growth (Supplementary Figure S4A) and the remainder grew similar to WT *in vitro*. Eight *FDL2.2* events, including four showing less growth and four WT-like events were transferred in soil. After growing in the LD greenhouse for 4 months, the *FDL2.2rd* transgenics were significantly shorter than WT plants (Figure 2D).

**Figure 3:**
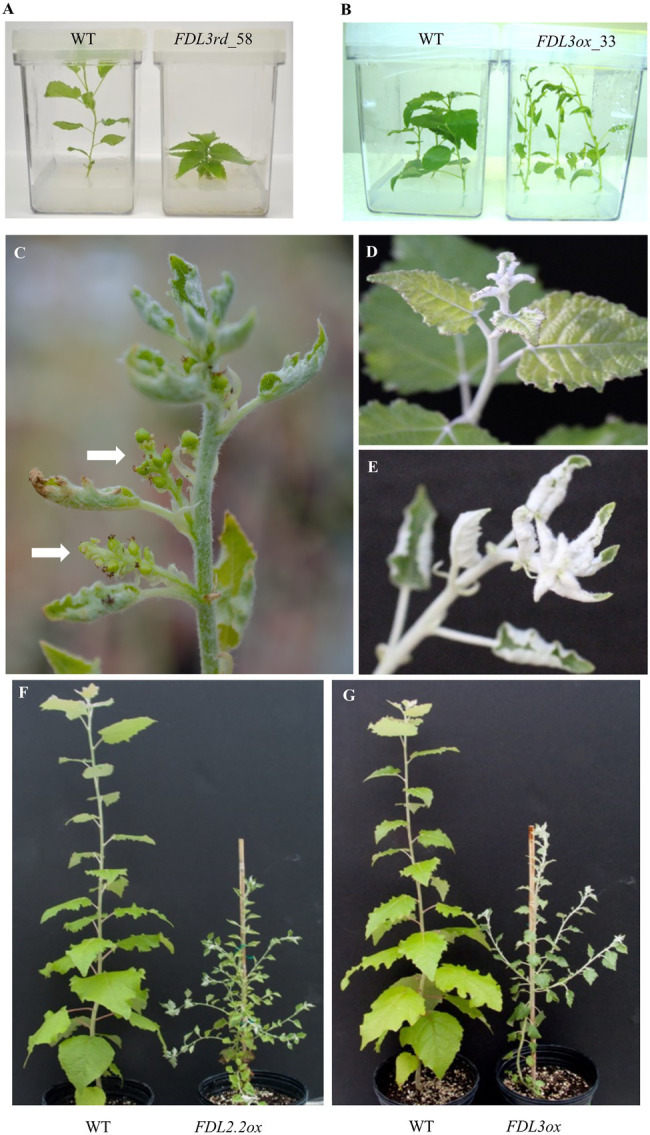
Overexpression of *FDL3* affects leaf size and shoot elongation but does not promote flowering. Representative plants showing opposite effects on shoot elongation *in vitro* of **(A)** *FDL3* dominant repression (*FDL3rd*) vs. **(B)** overexpression (*FDL3ox*). In **(A)** WT plant is 6-week-old, whereas *FDL3rd* plant is 10-week-old and in **(B)**, WT and *FDL3ox* were propagated at the same time and are 4-week-old in the photo. Shoot apices of **(C)** *FDL2.2ox* with consecutive axillary inflorescences, **(D)** WT, and **(E)** *FDL3ox*. White arrows in **(C)** point to axillary inflorescences with multiple female flowers. **(F)** A premature flowering *FDL2.2ox* plant with many inflorescences as shown in **(C)** on both the main shoot and branches. **(G)** A *FDL3ox* plant with a few branches, but no flowers. **(C–G)** All photos are of 6-month-old plants grown at the same time in a greenhouse under a 16-h photoperiod.

Whereas many *FDL3rd* transgenic shoots regenerated from callus, rooting was achieved for only five of these, possibly due to severe suppression of shoot elongation by the *FDL3rd* transgene (Figures 2B, 3A; Supplementary Figure 4B), and nearly all attempts to propagate these rooted shoots *in vitro* and acclimate *FDL3rd* transgenics to soil were unsuccessful. Ultimately, we were able to establish only a few ramets of two *FDL3rd* events in soil. After 2 months in a LD growth chamber, all three ramets of event *FDL3rd_56* grew to a height of only 10 cm or less and set terminal buds, as opposed to WT plants which reached 40 cm–50 cm in height and maintained active SAMs (Figure 2B). Two ramets of event *FDL3rd_52* survived after transfer to soil and showed reduced height growth compared to WT (Figure 2C). In sum, all of the dominate repressor versions of the different poplar *FDL* genes reduced shoot elongation, but their effects varied in magnitude with *FDL1rd* > *FDL3rd* > *FDL2.2rd*.

### *FDL2.2* but Not *FDL3* Induces Early Flowering Under LDs

We produced transgenics with the 35S promoter directing expression of *FDL2.2* or *FDL3*, designated *FDL2.2ox* and *FDL3ox*, respectively. Sixteen independent events each of *FDL2.2ox* and *FDL3ox* were regenerated. A previous study showed that *FDL2.2* overexpression transgenics had small leaves and flowered *in vitro* (Parmentier-Line and Coleman, 2016). We observed a similar *FDL2.2ox* phenotype (Supplementary Figure S4C); however, we only observed *in vitro* flowering on one *FDL2.2ox* plant. We consistently observed that, in contrast to *FDL3rd* plants that were much shorter than WT, *FDL3ox* shoots elongated faster with longer internodes than WT plants propagated at the same time (Figures 3A,B). Because *FDL2.2ox* and *FDL3ox* transgenics had similar small leaf phenotypes *in vitro*, we directly compared the ability of the two transgenes to induce flowering under LDs. Three to five ramets of five events of *FDL2.2ox* and five events of *FDL3ox* were propagated and transferred to soil in parallel with WT plants. The potted plants were grown in the greenhouse under LDs. All transgenics had small leaves as was the case *in vitro* and also exhibited branching, especially the *FDL2.2ox* transgenics (Figures 3F,G). Transgenics were also shorter than WT, which was not the case for *FDL3ox* plants *in vitro* where sucrose is provided in the medium (Figure 3B).

Within 6 months of growth in the greenhouse, all ramets of all *FDL2.2ox* events flowered. Transgenics formed consecutive axillary inflorescences and terminal inflorescences also formed on some of the plants (Figure 3C). However, we did not observe flowering on any of the *FDL3ox* plants (Figure 3E). In addition, two of the *FDL3ox* events were grown for an additional 10 months with no flowering. Thus, whereas *FDL2.2* or *FDL3* overexpression induces similar vegetative phenotypes in LDs (Figures 3F,G), they are not equivalent in their ability to induce flowering. This is also consistent with their different expression patterns, particularly the high *FDL2.2* expression in newly initiated reproductive buds (Figure 1D). *FDL2.2* overexpression has been previously studied (Parmentier-Line and Coleman, 2016); hence, we focused on further characterization of *FDL3ox* transgenics where vegetative phenotypes could be studied without confounding effects of precious flowering.

### Overexpression of *FDL3* Accelerates Leaf Initiation but Delays Leaf Expansion and the Transition to Secondary Growth Under LDs

**Figure 4:**
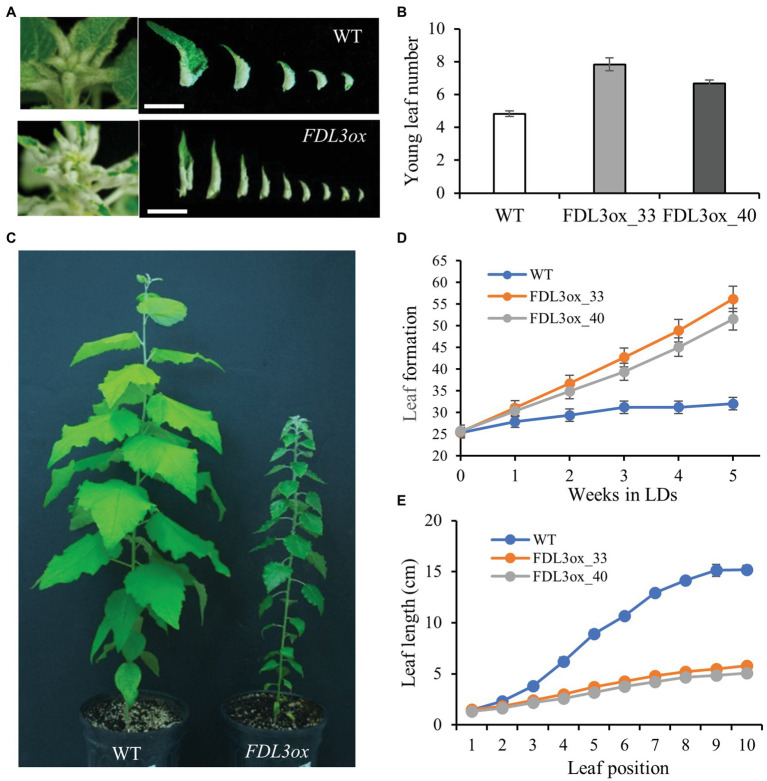
Overexpression of *FDL3* accelerates leaf production, but represses leaf growth in long day (LD) conditions. Ramets of two *FDL3ox* events and WT were grown in a growth chamber under 16-h photoperiods for 2 months. **(A)** Shoot apices and young rolled leaves of a *FDL3ox* plant compared with that of a WT plant. Scale bars = 1 cm. **(B)** Number of young rolled leaves. **(C)** Representative WT and *FDL3ox* trees **(D)** Emergence of new leaves (leaf lamina longer than 1 cm) over time. Leaf number was counted weekly, beginning 3 weeks after transplantation. **(E)** Progression of leaf length with position on the shoot. Leaf position 1 is the youngest leaf whose lamina is longer than 1 cm. **(B,D,E)** Means ± SE (*n* = 6) for two *FDL3ox* events (33 and 40) and WT.

**Figure 5:**
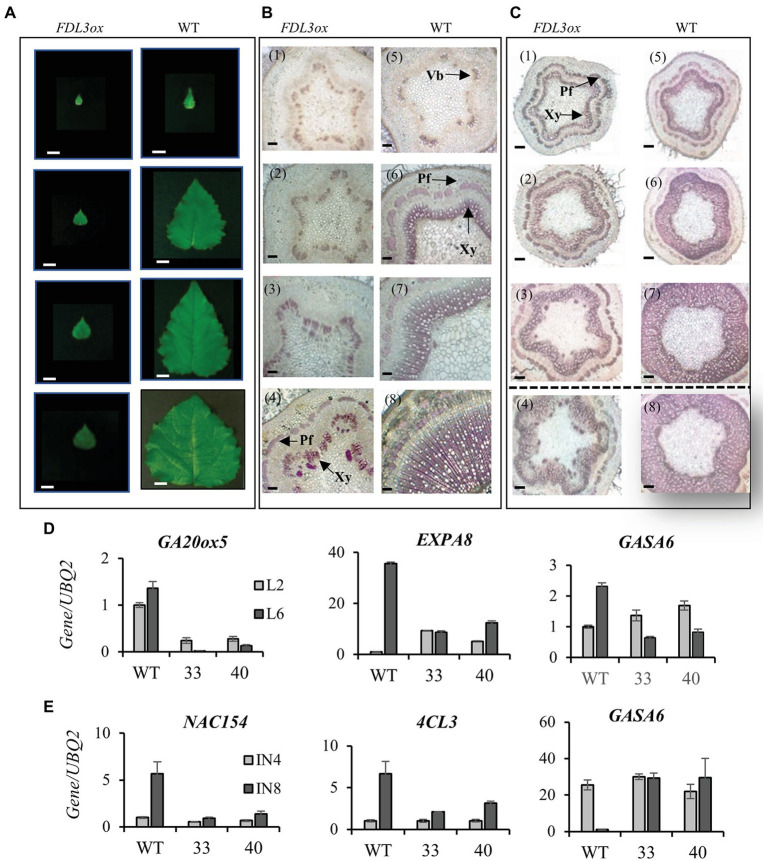
Overexpression of *FDL3* synchronously inhibits leaf expansion and the transition to secondary growth in long days (LDs), but secondary growth is restored in short days (SDs). **(A–C)** Both *FDL3ox* and WT plants were grown in a LD greenhouse for 6 months and subsequently transferred to a SD growth chamber for 8 weeks. Leaves were counted from top to bottom according to leaf plastochron index (LPI). Internode (IN) number refers to the internode beneath the corresponding LPI. All panels show from top to bottom LPI2, LPI4, LPI6, and LPI10 leaf or corresponding IN. **(A)** Extremely slow growth of *FDL3ox* leaves compared to WT in LDs. Scale bars = 2 cm. **(B)** Severely inhibited secondary growth in *FDL3ox* plants **(B1–4)** compared to progressive transition to secondary growth in WT **(B5–8)** in LDs. **(C)** Secondary growth in IN2, IN4, and IN6 formed after exposure to SDs (images above dotted line) in *FDL3ox* **(C1–3)** and WT **(C5–7)** plants. Note that *FDL3ox* INs 4 and 6 **(C2,3)** now resemble the same INs of WT plants grown in LDs **(B6,7)**. In contrast, IN10 **(C4)** formed in LDs before SD treatment remained underdeveloped in *FDL3ox*. After exposure to SDs, WT plants ceased elongation growth, IN2 transitioned to secondary growth **(C5)** and substantial secondary xylem accumulated in IN4 and IN6 of WT **(C6,7)**. Transverse sections were 60 μm thick, Scale bars = 100 μm. Vb, vascular bundles; Pf, phloem fiber; and Xy, xylem. **(D,E)** Comparative expression analysis of leaf and stem developmental marker genes in WT and two events of *FDL3ox* (33 and 40) grown in LDs. Relative expression in LPI2 and LPI6 leaves **(D)** and in internodes IN4 and IN8 **(E)**. Expression was normalized against reference gene ubiquitin gene (*UBQ2*).

We found that despite their reduced height growth compared to WT, potted *FDL3ox* transgenics initiated phytomers more rapidly, indicated by the formation of new leaves over time (Figure 4D). In contrast, *FDL3ox* leaf expansion progressed much more slowly and fully expanded leaf size was reduced (Figure 4). In addition to stalled leaf development, *FDL3ox* trees had vine-like stems that were not self-supporting (Figure 3G; Supplementary Figure S5A), suggesting defects in secondary vascular tissue development. Under LDs, primary growth of poplar shoots is limited to the leaf development zone and the transition from primary to secondary growth occurs below a leaf that is at least partially mature (Larson and Isebrands, 1971, 1974). Detailed study of leaf and stem development in two *FDL3ox* events illustrates the coordinate delay in leaf expansion and transition to secondary growth (Figures 5A,B). Vascular development in IN4 and IN6 of *FDL3ox* (Figures 5B2,B3; Supplementary Figure S6) remained nearly the same as in IN2, with the exception that red-stained lignified cells were present at the position where secondary xylem would normally develop in IN6. Additionally, no phloem fiber bundles were formed in either IN4 or IN6 (Figures 5B2,B3; Supplementary Figure S6), whereas transitional secondary growth in IN4 and secondary growth in IN6 was evident in the stem of WT plants (Figures 5B6,B7; Supplementary Figure S6). Phloem fiber bundles were present in IN10 of *FDL3ox*, but the secondary xylem still remained in a much less developed state compared to WT (Figures 5B4,B8; Supplementary Figure S6). Secondary xylem was still very poorly developed at IN20 of *FDL3ox* plants, and while increased xylem development was evident in IN30, it was not present in a continuous ring (Supplementary Figures S5B,5C, S6).

Little is known about the mechanisms coordinating the timing of leaf maturation and the transition to secondary growth (reviewed in Teixeira et al., 2019); however, GA promotes both leaf expansion and secondary growth (Eriksson et al., 2000). Hence, we studied the expression of GA synthesis and response genes that also show developmentally responsive expression changes. *GA20-oxidase 5* (*GA20ox5*) expression increases as leaves develop, and its expression was reduced in both LPI2 and LPI6 leaves of *FDL3ox* trees compared to WT (Figure 5D). GA3 application elevates *α-EXPANSIN 8* (*EXPA8*) and *GA-STIMULATED ARABIDOPSIS 6* (*GASA6*) expression in *Populus* leaves (Xie et al., 2016). In WT, *EXPA8* and *GASA6* are upregulated in LPI6 compared to LPI2 leaves, but not in *FDL3ox* plants (Figure 5D). In Arabidopsis, GA promotes *GASA6* upregulation and elongation in embryos (Zhong et al., 2015), and in the inflorescence stem, *GASA6* expression peaks in regions undergoing maximal elongation (Hall and Ellis, 2013). In *Populus* stems, *GASA6* is most highly expressed in internodes undergoing maximal elongation and downregulated in secondary growth internodes (Dharmawardhana et al., 2010). Whereas *GASA6* is downregulated in secondary growth IN8 compared to elongating IN4 in WT, it shows no downregulation in *FDL3ox* IN8 (Figure 5E). Expression of marker genes for secondary xylem further supports that molecular programs for the transition to secondary growth are not initiating properly in *FDL3ox* trees. *NAC154*, a co-ortholog of *SECONDARY WALL-ASSOCIATED NAC DOMAIN 2* (Grant et al., 2010), and *4-COUMARATE:COA LIGASE 3* (*4CL3*; Shi et al., 2010), are several fold higher in IN8 compared to IN4 in WT, but show comparatively low expression in IN8 of *FDL3ox* trees (Figure 5E).

### SDs Restore Leaf Expansion and Secondary Growth of *FDL3ox* Plants

**Figure 6:**
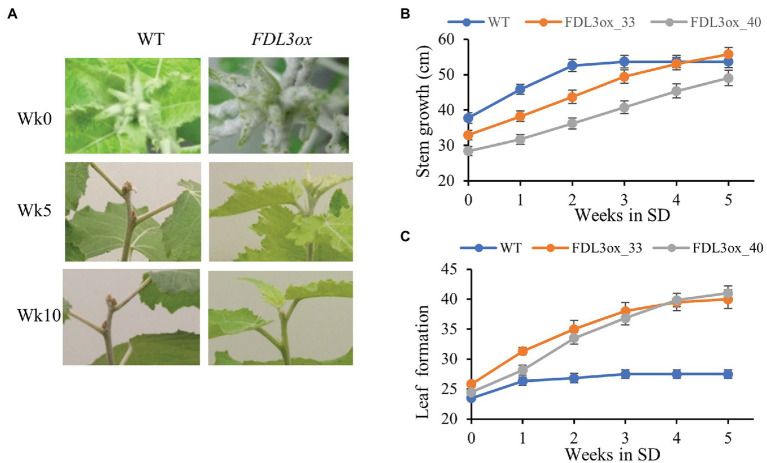
Overexpression of *FDL3* delays growth cessation and bud set in short days. Plants of WT and two *FDL3ox* events (33 and 40) were grown in long days for 2 months before exposure to SDs. **(A)** Apical bud development of *FDL3ox* plants compared to WT after 5 and 10 weeks in SD (Week 5 and Week 10). In Week 5, WT plants had formed buds. In contrast, *FDL3ox* plants maintained actively growing apex. By Week 10, *FDL3ox* plants formed buds. **(B,C)** Cumulative stem growth **(B)** and leaf formation **(C)** were measured weekly during the first 5 weeks in SDs. **(B)** Plant height and **(C)** leaf numbers are means ± SE (*n* = 6).

After 3 weeks of exposure to SDs, height growth and formation of new leaves stopped in WT plants, whereas *FDL3ox* plants showed no sign of growth cessation (Figures 6B,C). After 5 weeks in SDs, WT plants had formed brown apical buds, while shoot apices of *FDL3ox* plants remained active; *FDL3ox* plants eventually formed apical buds after 10 weeks in SDs (Figure 6A).

**Figure 7:**
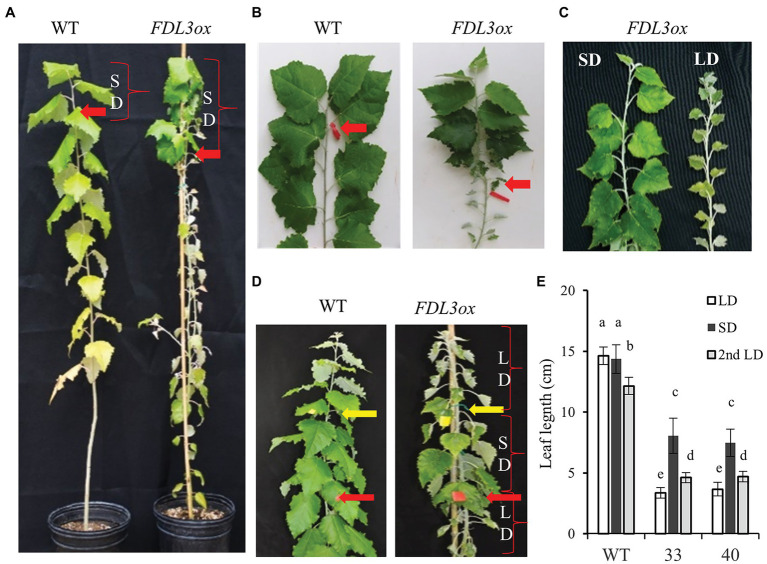
*FDL3ox* trees resume leaf development in short days. **(A,B)** Both *FDL3ox* and WT plants were grown in a long day (LD) greenhouse for 6 months and then were transferred into a SD growth chamber for 8 weeks. *FDL3ox* leaves formed after transfer to SDs (above the red arrows) showed leaf development similar to actively growing WT plants, in contrast to underdeveloped leaves formed on *FD3ox* plants in LDs (below the red arrows). Plants **(A)** and shoots **(B)** were imaged after 8 weeks exposure to SDs. **(C)** Shoots from ramets of the same *FDL3ox* event grown 8 weeks in SDs or LDs. **(D,E)** The changes in leaf expansion size of *FDL3ox* plants followed the changes of photoperiod duration. **(D)** *FDL3ox* and WT plants were grown for 2 months in LDs (below the red arrows), followed by 4 weeks of SDs (between red arrows and yellow arrows), and then 3 weeks of LDs (above the yellow arrows). **(E)** Fully expanded leaf length of WT and *FDL3ox* plants formed in SDs and LDs. Six fully expanded leaves were measured for each plant. Leaf length is mean ± SE (*n* = 4); different letters indicated significant differences, *p* < 0.0001, Tukey–Kramer’s test.

Intriguingly, *FDL3ox* plants not only continued to grow in SDs, but also the development of leaves formed after transfer to SDs was similar to that of leaves of WT grown in LDs (Figures 7A–C). In WT plants, leaves directly below the forming apical bud and formed under LDs continued to expand after transfer to SDs (Figures 7A,B). In contrast, small *FDL3ox* leaves formed during LD treatment did not increase in size during SD treatment. Strikingly, *FDL3ox* leaves that formed after transfer from LDs to SDs expanded rapidly, exceeding the length of those produced under LDs by ~2-fold. The return of SD-treated *FDL3ox* plants to LDs again led to the production of the small leaves like those produced during the first LD treatment (Figures 7D,E). We confirmed that the effect of *FDL3ox* overexpression on leaf development is dependent on photoperiod alone and not location (i.e., moving plants from greenhouse to growth chamber) by growing plants entirely in growth chambers with only photoperiod altered. Within 4 weeks in SDs, leaf expansion and height of *FDL3ox* were the same as WT plants that had started to form apical buds (Supplementary Figures S7B,C). In contrast, *FDL3ox* plants grown in LD conditions were shorter with small leaves (Supplementary Figures S7A,C).

The SD-mediated restoration of leaf expansion in *FDL3ox* (Figure 7B), prompted us to examine secondary growth of the stem segments formed under SD conditions. After 8 weeks in SD conditions, substantial secondary xylem and phloem fiber bundles were present in IN2 of WT plants, which set bud before the internode was collected (Figure 5C5). In contrast to the poor secondary growth of *FDL3ox* plants under LD conditions (Figures 5B1–4), a closed circle of secondary xylem and phloem fiber bundles were present in all three internodes of *FDL3ox* plants formed under SD (Figures 5C1–3). Similar to leaves of *FDL3ox* plants formed under LDs and their failure to expand and mature under SDs (Figures 7A,B), the secondary xylem of IN10 formed in *FDL3ox* plants grown under LDs remained poorly developed in SDs (Figure 5C4).

### *FDL3ox* and Daylength Alter *FT2* and *AP1/FUL* Expression

The rapid change in *FDL3ox* leaf development following the shift from LD to SD conditions (Figure 7) prompted us to study changes in expression of *FT2* and possible transcriptional targets of *FDL3*. In rice, FT-FD complexes form in leaves as well as in SAM and the small leaf phenotype of *35S::FT* Arabidopsis under SDs requires *FD* and ectopic expression of *FUL* (Teper-Bamnolker and Samach, 2005; Brambilla et al., 2017). In source leaves, *FT2* is rapidly downregulated in response to SDs (Böhlenius et al., 2006; Hsu et al., 2011; Supplementary Figure S11) and studies suggest that *LIKE-AP1a* (*LAP1a*) acts downstream of a FT-FDL1 complex (Azeez et al., 2014; Tylewicz et al., 2015). Moreover, *Populus AP1/FUL* family members were upregulated in *35S:FDL2.2* transgenics (Parmentier-Line and Coleman, 2016), and inducible *FT1* or *FT2* expression upregulated *FUL* expression (Hsu et al., 2011).

**Figure 8:**
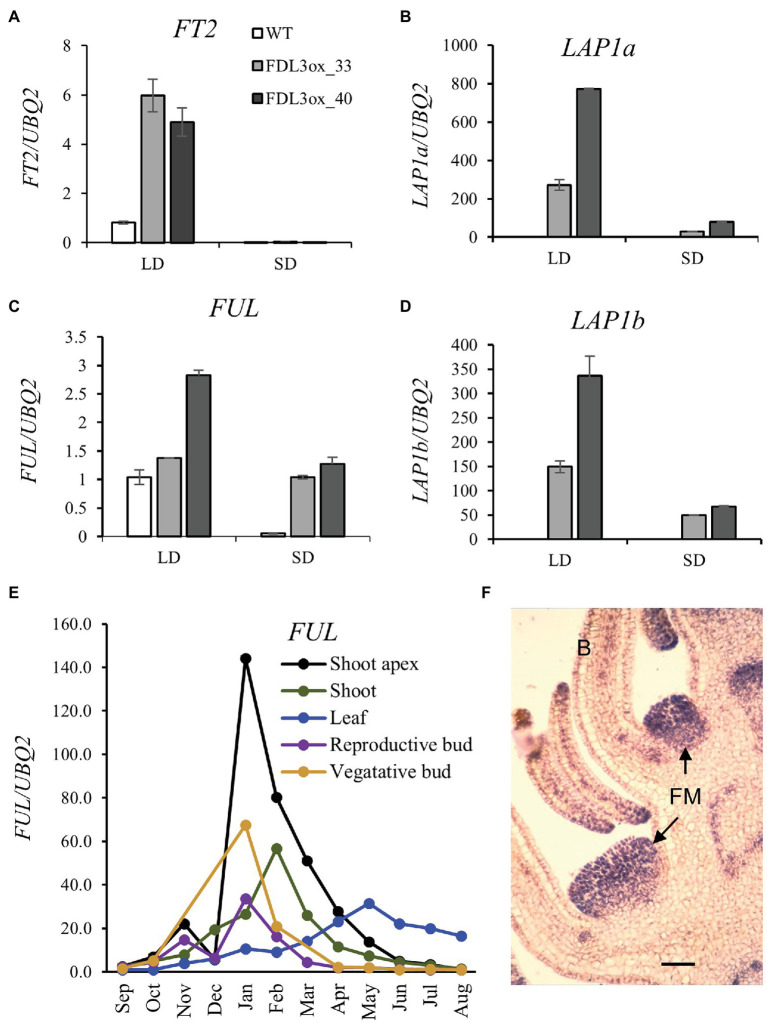
*FDL3ox* and daylength alter expression of *FT2* and three *AP1/FUL* homologs with diverse vegetative and reproductive expression patterns. **(A–D)** Fully expanded leaves were collected from WT and two independent events of *FDL3ox* plants grown for 2 months in LDs, followed by 3 weeks in SDs. Relative fold changes in transcript levels of *FT2* **(A)**, *LAP1a* **(B)**, *FUL* **(C)**, and *LAP1b* **(D)**. Expression of the other two members of the *AP1/FUL* family (Supplementary Figure 8), *MADS14* and *MADS28*, was not detectable in either LDs or SDs. The expression was normalized against reference gene *UBQ2*. **(E)** Seasonal expression pattern of *FUL* in adult *Populus deltoides*. Relative expression is fold change in transcript levels relative to the time point with the lowest expression within a tissue (*n* = 3 biological replicates except that three technical replicates were assayed from a pool of three shoot apices). **(F)** *In situ* hybridization showing *LAP1b* expression in initiating floral meristems of an immature male *Populus trichocarpa* inflorescence. FM, floral meristem; B, bract. Scale bar = 100 μm. Additional *LAP1a* and *LAP1b in situ* hybridizations are provided in Supplementary Figure S12.

The *P. trichocarpa* genome contains five members of the *AP1/FUL* family (Supplementary Figure S8A). In *FDL3ox* transgenics, only *LAP1a*, *LAP1b*, and *FUL* were upregulated under LDs and their expression levels were correlated (*R*^2^ ≥ 0.79) with expression level of the *FDL3ox* transgene (Supplementary Figure S8). In WT trees under LDs, *FT2* was dramatically upregulated as leaves near full expansion (LPI6), but leaf stage had comparatively little effect on *FDL3* expression (Supplementary Figure S9). Compared to WT plants, *FT2* expression in LPI6 leaf was 4–6-fold higher in *FDL3ox* transgenics in LDs (Figure 8A). In WT plants under LDs, *LAP1a, b* transcripts were low to barely detectable in shoot apices and LPI6 leaf, whereas *FUL* was relatively highly expressed and downregulated in multiple tissues in response to SDs (Supplementary Figure S10). However, all three genes were upregulated in LPI6 leaf and shoot apices of *FDL3ox* trees under LDs (Figures 8B–D, Supplementary Figure S12). In SDs, expression of *FT2* and the *AP1/FUL* homologs was reduced in both WT and *FDL3ox* leaf and/or shoot apex (Figures 8A–D, Supplementary Figure S11). These results suggest that under LDs, *FDL3ox* transgenics could have elevated levels of a FT2-FDL3 complex in leaves as well as shoot apices that activates *AP1/FUL* homologs but that under SDs, reduced *FT2* levels limit complex formation and *AP1/FUL* expression.

To elucidate possible endogenous roles and diversification of the three *AP1/FUL* homologs, we studied their expression patterns. Because *FUL* was expressed in various tissues of juvenile trees (Supplementary Figure S10), we studied its circannual expression in adult *P. deltoides*. *FUL* was expressed in all tissues, being most highly upregulated in shoot apices in winter with expression declining to lowest levels after shoots had set terminal buds in summer (Figure 8E). In juvenile samples, expression of the *AP1* co-orthologs (*LAP1a* and *LAP1b*) was low compared to *FUL* expression (Supplementary Figure S10), and transcriptome profiling of poplar floral bud development indicated a role for *LAP1* in flowering (Chen et al., 2018). Thus, we studied the spatial expression patterns of the *LAP1* paralogs in developing reproductive buds. *In situ* hybridization of early developmental stages showed strong expression in initiating floral meristems (Figure 8F; Supplementary Figure S12), consistent with the conserved role of *AP1* orthologs in specifying floral meristem identity (Pabon-Mora et al., 2013).

## Discussion

### Both Regulatory and Protein Variation Likely Contribute to the Functional Diversification of *FDL* Paralogs

The different tissue and seasonal expressional patterns of poplar *FDL* genes indicate that regulatory diversification of *FDLs* is linked to distinct roles in flowering and vegetative phenology. *FDL2* is distinct in that it is the only *FDL* showing predominately reproductive expression (Figure 1). Both splice variants are upregulated in initiating axillary inflorescence buds, consistent with a canonical function in the control of the floral transition. However, only *FDL2.2* induces early onset of flowering (Figure 3; Tylewicz et al., 2015; Parmentier-Line and Coleman, 2016). *FDL1* expression was highest in late fall and winter (Figures 1A,E), consistent with one of its previously proposed functions, cold adaptation (Tylewicz et al., 2015). Conversely, *FDL3* is expressed in shoot apices during the growing season (Figures 1C,E) and *FDL3ox* plants initiated new leaves at a faster rate than WT under LDs (Figure 4D), suggesting that *FDL3* might be the paralog with a primary role in promoting apical growth.

The dominant repressor version of each of the *FDL* genes reduced shoot elongation in LDs to markedly different degrees (Figures 2, 3A; Supplementary Figure S4), indicating that the proteins share partial functional equivalency. In contrast, growth was extended in SDs by overexpression of *FDL1*, *FDL2.2*, or *FDL3* (Figure 6; Tylewicz et al., 2015; Parmentier-Line and Coleman, 2016), but detection of any differences in degree of this SD phenotype was not possible as each *FDL* transgenic was studied in a different lab. However, transgenics overexpressing WT versions also supported partial functional equivalency of the three FDL proteins because these transgenics differed in regard to other phenotypic effects. *FDL1* overexpression did not induce obvious changes in growth and development under LDs (Tylewicz et al., 2015), whereas overexpression of either *FDL2.2* or *FDL3* altered vegetative phenotypes, but only *FDL2.2* induced flowering (Parmentier-Line and Coleman, 2016; Figure 3).

The only difference between the proteins encoded by the two *FDL2* splice variants (*FDL2.1* and *FDL2.2*) is an additional 29 amino acids within the FDL2.1 bZIP domain (Supplementary Figure S1CS). *FDL2.2* promoted dramatic changes in vegetative phenotype and precocious flowering, while *FDL2.1* overexpression inhibited growth (Tylewicz et al., 2015), yielding a similar albeit weaker version of the growth suppression resulting from *FDL2.1rd* overexpression (Supplementary Figure S4). Perhaps the 29 additional amino acids in FDL2.1 interfere with transcriptional activation of FDL2 target genes, weakly mimicking the *FDL2.1rd* phenotype. That the seasonal fluctuations of *FDL2.1* and *FDL2.2* expression in the shoot apex and tissue-level expression patterns of these variants are similar (Figure 1; Supplementary Figure S3) also points to the possibility that *FDL2.1* acts as a regulator of *FDL2.2*.

With knowledge of the varied differences among the *FDL*-induced phenotypes, comparison of protein sequences can suggest domains to target for future analysis to determine the sequence variation responsible for protein functional divergence. For example, both *FDL2.2ox* and *FDL3ox* plants had small leaves and vine-like stems in LDs, but *FDL3ox* did not induce flowering (Figure 3). Tsuji et al. (2013) proposed that the LSL motif (Supplementary Figure S1C) is important for flowering as it was present in most eudicot FDs, but among diverse Poaceae FDs, it was limited to the subgroup containing flowering-promoting FDs. In FDL3, the first leucine of the LSL motif is replaced with serine, suggesting a candidate mutation for the absence of flowering-promoting activity.

The *FD*, *FT*, and *AP1/FUL* families have conserved roles in flowering, but studies in diverse angiosperms have also shown different patterns of gene duplication/loss and subsequent functional evolution (Litt and Irish, 2003; Abe et al., 2005; Wigge et al., 2005; Hsu et al., 2011; Taoka et al., 2011; Tsuji et al., 2013; Tylewicz et al., 2015). Whereas the two *Populus FT*s derive from the Salicoid WGD, the *FD* and *AP1/FUL* families are more complex, with Salicoid duplicates retained for only some members (*FDL1/FDL2* and *LAP1a/LAP1b*; Rodgers-Melnick et al., 2012). Similar to the *FT* paralogs, *FDL1* and *FDL2* show highly divergent expression patterns (Figure 1; Hsu et al., 2011). Whereas it remains to be determined if this allowed *FT* and *FDL* duplicates to evolve in concert, the overlap in seasonal peak expression (winter) for *FT1* and *FDL1* (Figure 1A; Hsu et al., 2011) and that their proteins interact (Tylewicz et al., 2015) suggest this as a possibility. *FT1*, *FDL2.2*., and *FT2*, albeit less effectively, induce precocious flowering when overexpressed (Figure 3; Böhlenius et al., 2006; Hsu et al., 2006, 2011; Parmentier-Line and Coleman, 2016). In contrast, overexpression of *FDL1* or *LAP1a* did not induce early flowering (Azeez et al., 2014; Tylewicz et al., 2015), but insufficiency does not preclude a role in flowering. *FT1* is upregulated in winter vegetative buds, and detailed study of reproductive development indicated that inflorescence buds subsequently (i.e., shortly after bud flush) develop in the axils of late preformed leaves (Yuceer et al., 2003; Hsu et al., 2011). Although *FT2* is predominately expressed in source leaves, it is also expressed coincident with *FDL2.2* in developing spring inflorescence buds (Figure 1D; Hsu et al., 2011) and *LAP1a* and *LAP1b* are expressed in initiating floral meristems (Figure 8F, Supplementary Figure S12). We previously posited that both *FT1* and *FT2* could have roles in flowering but act at different stages—*FT1* might promote the transition of incipient axillary meristems within winter buds to inflorescence meristems, while *FT2* might promote floral meristem initiation within the developing inflorescence (Brunner et al., 2014). Perhaps *FDL1* and *FDL2* could have similarly diversified roles in flowering as well as the suggested diversification of three *FDLs* in vegetative development. Gene editing can potentially clarify their gene-specific functions; however, given the difficulties in generating plants with *FDL1rd* or *FDL3rd* transgenes, this might require mutations that reduce or alter only specific sequences/functions rather than knock out gene activity. Use of an *FT*-mediated early flowering poplar system (Azeez and Busov, 2019) could help delineate roles in flowering. Moreover, *FDL2* is potentially a candidate for CRISPR-mediated manipulation to prevent flowering and mitigate gene flow from plantations. For forest trees, not only both male and female reproductive sterility but prevention of flower formation is desired for biosafety (Fritsche et al., 2018).

### *FDL3* Overexpression Induces Heterochronic Shifts in Shoot Ontogeny Depending on Photoperiod

In addition to a faster rate of phytomer initiation in LDs, leaf expansion and the transition to secondary growth were greatly delayed in *FDL3ox* shoots (Figures 4, 5; Supplementary Figures S5, S6). The formation of a vascular cambium introduces another sink; thus, the occurrence of this transition at a distance from an active SAM and below a leaf that is at least partially mature reflects sink-source relationships (Larson and Isebrands, 1971, 1974). Moreover, the direction of carbon transport changes as leaves develop (Dickson and Larson, 1981; Isebrands and Nelson, 1983). In general, transitional leaves transport carbon upward to younger leaves and SAM, recently mature leaves transport in both directions, and older leaves transport carbon to the lower stem and roots. The delayed transition to secondary growth in *FDL3ox* plants (Figure 5B; Supplementary Figure S6) might be a direct consequence of stalled leaf development preventing production and transport of sufficient sugar to initiate and support secondary growth. A more active SAM (i.e., more rapid phytomer initiation; Figure 4D) and hence increased SAM sink strength in *FDL3ox* plants might further limit sugar import for leaf expansion and concurrently, reduce carbon availability for cambium formation. *FT2* upregulation is strongly correlated with leaf maturation (Supplementary Figure S9B). In Arabidopsis, the signaling sugar trehalose-6-phosphate (T6P) increases *FT* expression in leaves to promote florigen (Wahl et al., 2013). Thus, it will be interesting to determine if the T6P pathway acts to induce *FT2* expression in source leaves, linking *FT2* signaling to leaf sugar status and export direction to apical and cambial meristems.

Overexpression of *GA20ox* in poplar increased leaf size, height and diameter in LDs and delayed SD-induced growth cessation but not *FT2* downregulation, suggesting that *FT2* and GA act in parallel growth-promoting pathways (Eriksson et al., 2000, 2015). In *FDL3ox* trees, reduced *GA20ox5* expression in leaves and that GA-responsive genes *GASA6* and *EXPA8* were not upregulated suggests that reduced GA levels and signaling contributed to reduced leaf expansion (Figure 5D). In tobacco, GA signaling from maturing leaves is important for both shoot elongation and radial growth (Dayan et al., 2012) and GA can act systemically to delay SD-induced growth cessation in poplar (Miskolczi et al., 2019). However, the *FDL3ox* phenotype does not support that reduced GA levels in leaves reduces apical growth as *FDL3ox* trees initiate phytomers at a faster rate and SD-induced growth cessation is delayed (Figures 4D, 6). Moreover, *FDL3ox* plants grew faster with longer internodes than WT *in vitro* on sugar-containing media, the opposite of *FDL3rd* plants (Figures 3A,B). This suggests that the reduced height growth of potted *FDL3ox* plants (Figures 3G, 4C) could be a secondary effect of small transgenic leaves providing less photosynthate than WT leaves.

Gibberellin is synthesized in apices and signaling might be predominately local as grafting onto GA-overexpressing rootstocks was less effective at delaying SD-induced growth cessation in WT scions than grafting onto *FT*-overexpressing rootstocks (Miskolczi et al., 2019). Expression of stem elongation marker gene *GASA6* was similarly high in WT and *FDL3ox* IN4, but unlike in WT, showed no decrease in IN8 of *FDL3ox* trees, consistent with their protracted primary growth phase (Figure 5E). Although GA promotes wood formation, *GASA6* expression in *FDL3ox* stems suggests that a localized attenuation of GA-promoted stem elongation might be necessary for internodes to transition to secondary growth.

A striking phenotype of *FDL3ox* trees was the rapid change in leaf and stem development with photoperiod (Figure 7), indicating that effect of *FDL3* on shoot ontogeny depends on genes whose activity is controlled by daylength. In LDs, *FT2*, *LAP1*, and *FUL* expression is elevated in *FDL3ox* trees, but as in WT, all are downregulated by SDs (Figure 8; Supplementary Figure S11). However, in SDs, *LAP1* and *FUL* expression is still higher in *FDL3ox* plants compared to WT. This suggests that *FDL3* overexpression could increase the level of a FDL3-FT2 complex in leaf as well as SAM under LDs, elevating the expression of downstream targets to promote SAM activity, but limiting leaf expansion and the transition to secondary growth. Under SDs, perhaps reduced *LAP1/FUL* expression is sufficient to maintain SAM activity, but not to delay leaf development and the transition to secondary growth. However, elevated *LAP1a* expression is not sufficient to explain the *FDL3ox* phenotype as it did not alter leaf development (Azeez et al., 2014). Various FT and FD homologs have been shown to interact with other proteins (Mimida et al., 2011; Tsuji et al., 2013; Tylewicz et al., 2015; Jung et al., 2016; Brambilla et al., 2017; Li et al., 2019); thus, the effect of *FDL3* on leaf development and secondary growth could be independent of *FT2*. Heterochronic mutants have helped reveal genetic mechanisms controlling seed maturation and vegetative phase change (reviewed in Buendia-Monreal and Gillmor, 2018). Thus, further study of *FDL3ox* transgenics could provide an inroad into understanding the genetic pathways that link leaf and stem ontogenies.

## Data Availability Statement

The datasets presented in this study can be found in online repositories. The names of the repository/repositories and accession number(s) can be found in the article/**Supplementary Material**.

## Author Contributions

XS, C-YH, and AB designed experiments, performed research, and analyzed results. CM produced overexpression transgenics used in this study. XS and AB drafted the paper. C-YH and CM revised and provided comments on the manuscript. All authors contributed to the article and approved the submitted version.

## Funding

This study was supported by the US Department of Energy Office of Science, Office of Biological and Environmental Research, Grant No. DE-SC0012574 and the USDA National Institute of Food and Agriculture, McIntire Stennis project 1025004.

## Conflict of Interest

The authors declare that the research was conducted in the absence of any commercial or financial relationships that could be construed as a potential conflict of interest.

## Publisher’s Note

All claims expressed in this article are solely those of the authors and do not necessarily represent those of their affiliated organizations, or those of the publisher, the editors and the reviewers. Any product that may be evaluated in this article, or claim that may be made by its manufacturer, is not guaranteed or endorsed by the publisher.
