# Supplementary material for: Functional Diversification of Populus FLOWERING LOCUS D-LIKE3 Transcription Factor and Two Paralogs in Shoot Ontogeny, Flowering, and Vegetative Phenology
Source: Front Plant Sci. 2022 Feb 3;13:805101. doi: 10.3389/fpls.2022.805101 (PMC8850916; doi:10.3389/fpls.2022.805101)
Supplement: Supplementary file 1 [file Data_Sheet_1.PDF]

Supplementary Material

The following supplementary figures and tables are included in this document:

Supplementary Figure S1. Relationships of *FD* homologs.

Supplementary Figure S2. Alignment of 3' regions of *Populus AP1/FUL* family members.

Supplementary Figure S3. Expression of *FDL* genes in various tissues of 4-month-old *P. tremula* × *P. alba* clone 717-1B4 trees.

Supplementary Figure S4. Representative *in vitro* phenotypes of *FDL2.1, FDL2.2* and *FDL3* transgenics.

Supplementary Figure S5. *FDL3ox* plants show underdeveloped secondary growth.

Supplementary Figure S6. Micrographs comparing secondary growth transitions in WT and *FDL3ox* trees

Supplementary Figure S7. Effect of short daylengths on leaf size and height growth in young *FDL3ox* plants grown only in growth chambers.

Supplementary Figure S8. Upregulation of three *AP1/FUL* family members is positively correlated with *FDL3ox* expression level.

Supplementary Figure S9. *FDL3* and *FT2* expression in leaves at different developmental stages.

Supplementary Figure S10. Comparison of *LAP1a*, *LAP1b* and *FUL* expression levels and time course of *FUL* downregulation in response to short days.

Supplementary Figure S11. *FDL3ox* and daylength alter *LAP1a*, *LAP1b* and *FUL* expression in shoot apices.

Supplementary Figure S12. Spatial expression patterns of *LAP1a* and *LAP1b* in developing inflorescences.

Supplementary Table S1. Primers used in this study.

Supplementary Table S2. List of sequences used in phylogeny shown in **Supplementary Figure S1A**.

Supplementary Table S3. List of sequences used in phylogeny shown in **Supplementary Figure S10A**.

Supplementary Table S4. Relative expression levels (mean fold change ± SD) of *FDL* transcripts plotted in Figure 1.

Supplementary Table S5. Relative expression levels (mean fold change ± SD) of *FUL* transcripts plotted in Figure 8E.

A

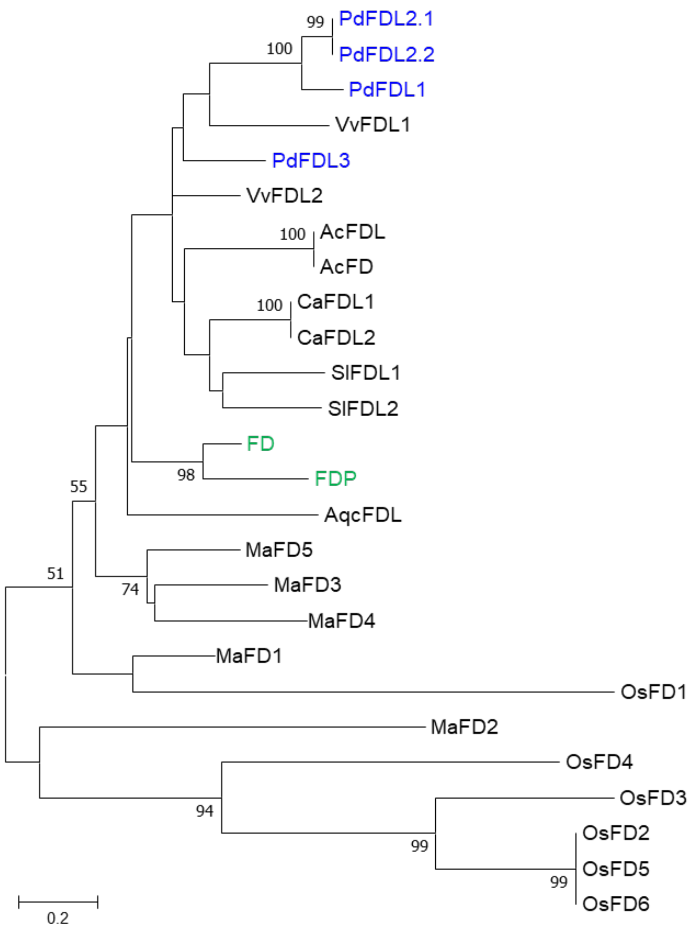

B

Sheng et al. (this paper)	Tylewicz et al. 2015	Parmentier-Line & Coleman 2016	Potri v3 or v4.1 as indicated	Podel v2.1	Differences between cDNAs and gene models and relationship to previous studies
FDL1	*FDL1*	*FD2*	Potri.002G018400.2 (v4.1)	Podel.02G017800.1	The proteins encoded by the *P. deltoides FDL1* cDNA and the Potri gene model are identical in length with 1 amino acid difference. The Podel model predicts different splicing and encoded protein is 26 aa shorter (lacks most of basic region). Compared to these 3 proteins, the FDL1 reported by Tylewicz et al. 2015 lacks 39 amino acids at the N-terminus.
FDL2.1	*FDL2*		Potri.005G243400.1 (v3.0)	Podel.05G259400.1	Splice variant studied by Tylewicz et al. 2015. All Potri v4.1 and Podel v2.1 transcripts differ from *FDL2.1* cDNA and listed Potri v3.0 model.
FDL2.2		*FD1*	Potri.005G243400	Podel.05G259400	Splice variant studied by Parmentier-Line & Coleman 2016; no corresponding transcript predicted in Potri v.3.0, v4.1 or Podel v2.1.
FDL3		*FD3*	Potri.005G109500.2 (v4.1)	Podel.05G122500.1	*P. deltoides* cDNA and Potri transcript differ from Podel predicted transcript that lacks region encoding 35 amino acids of the bZIP domain.

C

```
PdFDL1      -------------------MSTNKVSSNSPSKS-LYTCSSPSTLSSPSPIPNQSTTGNS-
PdFDL2.1    MWSSPGA------NIDNNNTSNSKVSGNSPSKCFSSTCSSPSPFSPSPPIPNQSMNGAS-
PdFDL2.2    MWSSPGA------NIDNNNTSNSKVSGNSPSKCFSSTCSSPSPFSPSPPIPNQSMNGAS-
PdFDL3      MLSPTDCEGT---SYNNKTKSLRKVSSSISSKS-SSTSSSPSIFSPSNNLHCQAQQKAE-
FD          MLSSAKHQRNHRLSATNKNQTLTKVSSISSSS--PSSSSSSSSTSSSSPLPSQDSQAQKR
FDP         ------------------------------------------------------------

                        A motif
PdFDL1      ----MEEVWNDINLASLHEH-----------PNSHTGSNNNTDDHVFH----GMMFQDLL
PdFDL2.1    ----MEEVWDDINLASLHDH-------------SNTNTSSNTNHHSFN----GMVFQDFL
PdFDL2.2    ----MEEVWDDINLASLHDH-------------SNTNTSSNTNHHSFN----GMVFQDFL
PdFDL3      ---TMEEVWKDISLASLHDHTSTDQELSMTPRLHNISHHHRHHHHHHNNNGPNFILQDFL
FD          SLVTMEEVWNDINLASIHHL-------NRHSPHPQHNHEPRFRGQNHHNQNPNSIFQDFL
FDP         ----MEEVWKEINLGSLHYH-----------RQLNIGHEPMLKNQNPN----NSIFQDFL
                *****.:*.*.*:*                : .       :  :    . ::**:*

                                                       LSL motif
PdFDL1      ARSSNKDTPTRVAS--KEPSSGGGNNFLKNSLG----PPPATMLNLNYG-----------
PdFDL2.1    ARPSNKDTSTRAAS--KEPSSGGGNSFLKKSLG----PPPATMLSLNSGSDHFHYLESSD
PdFDL2.2    ARPSNKDTSTRAAS--KEPSSGGGNSFLKKSLG----PPPATMLSLNSGSDHFHYLESSD
PdFDL3      ARPFNKDPPTRMVSIIRDSTPFGSSA-----------PPPATVSSLNSGPG-FDFLENSD
FD          KGSLNQEPAPTSQT--TGSAPNGDSTTVTVLYSS-PFPPPATVLSLNSGAG-FEFLDNQD
FDP         NMPLNQPPPP--------PPPPSSSTIVTALYGSLPLPPPATVLSLNSGVG-FEFLDTTE
              . *: ...        ... ...            *****: .** *

PdFDL1      -------------------------------------------KRPQENGDVSGGDRRHE
PdFDL2.1    TVPVRPNPQMHSHANGGTISFDSSLDSPFDALGSSSAFLSICKKRPQENGDVSGGDRRHK
PdFDL2.2    TVPVRPNPQMHSHANGGTISFDSSLDSPFDALGSSSAFLSICKKRPQENGDVSGGDRRHK
PdFDL3      H-PQRPDSQLQSNP----ISNISSFTSPFEGLDSSPGLPSFCKKRTQESDGSS-GDRRHK
FD          P-LVTSNSNLHTHHH---LSNAHAFNTSFEALVPSS---SFGKKRGQDSNEGS-GNRRHK
FDP         N-LLASNPR------------------SFEESAKFG---CLGKKRGQDSDDTR-GDRRYK
                                                       ** *:..    *:**::

            Basic region (bZIP domain)
PdFDL1      RMIKNRESAARSRARKQ-----------------------------AYTTELELKVALLG
PdFDL2.1    RMIKNRESAARSRARKQESSSPFENLFLVKFNDYRMLMFYLLLILQAYTVELEREAAHLA
PdFDL2.2    RMIKNRESAARSRARKQ-----------------------------AYTVELEREAAHLA
PdFDL3      RMIKNRESAARSRARKQ-----------------------------AYTNELENEVEQLL
FD          RMIKNRESAARSRARKQ-----------------------------AYTNELELEVAHLQ
FDP         RMIKNRESAARSRARKQ-----------------------------AYTNELELEIAHLQ
            *****************                             *** *** :   *

            Leucine-rich region (bZIP domain)   SAP motif
PdFDL1      EENAKLRKQQERFLAAAPAQPPKKHTLYRTSTAPF
PdFDL2.1    QENAKLRRQQERFLAAAPAQLPKKKTLYRTSTAPF
PdFDL2.2    QENAKLRRQQERFLAAAPAQLPKKKTLYRTSTAPF
PdFDL3      KENARLKRQQEELYLAAAAQLPKKHTLQRTSTAPF
FD          AENARLKRQQDQLKMAAAIQQPKKNTLQRSSTAPF
FDP         TENARLKIQQEQLKIAEATQNQVKKTLQRSSTAPF
             ***.*. **: :  * . *   *:** *:*****
```

D

>*PdFDL1*
cttcatctccagtcactccgagtcacatctcctctcgcatggcctcctgatccgtgtttccttgtaaaagtatgtgatcagtggcaggagataaaaatata
aataaaaacagcATGAGCACCAATAAAGTCTCTAGTAATTCTCCGTCAAAATCCCTCTATACATGTTCATCTCCTTCAAC
TCTGTCTTCTCCTTCCCCAATACCAAACCAATCAACGACTGGAAACTCAATGGAAGAAGTTTGGAATGATATAAACC
TAGCTTCTCTTCATGAACATCCAAATAGTCACACAGGCAGCAACAACAACACCGACGACCATGTTTTTCACGGTATG
ATGTTTCAAGACCTCTTGGCTAGATCTTCCAATAAAGACACACCAACAAGGGTTGCCTCTAAGGAACCCTCATCTG
GTGGGGGCAACAATTTCTTGAAGAACTCTTTAGGGCCGCCACCAGCTACTATGCTGAATTTGAATTATGGAAAAAG
GCCTCAAGAAAACGGTGACGTTTCTGGCGGCGATCGGAGGCATGAGCGCATGATCAAGAACAGAGAATCTGCAG
CTCGGTCCCGGGCTAGGAAGCAGGCTTACACGACCGAGTTGGAACTTAAAGTTGCTCTGTTAGGAGAGGAGAAT
GCCAAGCTTAGAAAGCAGCAAGAAAGGTTCTTGGCAGCAGCTCCTGCTCAGCCACCAAAAAAGCACACCCTCTAT
CGAACCTCAACGGCTCCATTTTGAgaagtaagaaatctatgcttagcccatttcatccctttatgtttccacttggctatcttgccaaaaacttc
cttgaaaagagggagatgttttccatatatcatttcaaagggcttttgatgtaaaagaaaggaggggaaaagagaggtaggaaagtagcgaggaca
aattaaagttgcctggacgccaagccgcaggaccagttgaggggtcgtattttattttcttgaaccactatttcttcactcctttgtcttcttttccatgtttg
ctttctaattttcctcctcctatgtggacgctgtgacgttgggagagataaatgccaagggaaaatagccttagcttgtttgtttgcctacaaatatgtat
ataaagatgggacctgtttggctatcaatgtcactttctattctaatcttatctattcattttcagtgtcctcctttccaaaaaaaaaaaaaaaaaaaaaa
aaaaa

>*PdFDL2.2*
cagaaagaaacatcatttcatctccagttactcctagtgctgttttccctgtcaaagcATGTGGTCATCGCCAGGAGCAAATATTGATAA
CAACAACACGAGCAACAGTAAAGTCTCTGGCAATTCTCCTTCAAAATGCTTTTCCTCTACATGTTCTTCTCCTTCACC
TTTCTCTCCTTCTCCTCCAATACCAAACCAATCAATGAACGGAGCCTCAATGGAAGAAGTTTGGGATGACATAAACC
TAGCTTCTCTTCATGATCATTCAAATACTAACACAAGCAGCAACACCAACCACCATTCTTTTAATGGTATGGTCTTTC
AAGATTTCTTGGCTAGACCTTCCAATAAAGACACATCAACAAGGGCTGCCTCTAAGGAACCCTCCTCTGGCGGGGG
TAACAGTTTCTTGAAGAAATCTTTAGGGCCACCCCCAGCTACCATGCTGAGTTTGAATTCTGGGTCTGATCATTTTC
ATTATCTGGAAAGCAGTGATACTGTCCCTGTGAGGCCAAATCCACAAATGCATAGTCATGCCAATGGTGGCACAAT
AAGTTTTGATTCTTCTCTCGATTCCCCCTTTGATGCCTTGGGTTCTTCTTCAGCGTTCCTTTCCATTTGCAAAAAAAG
GCCTCAAGAAAACGGTGATGTCTCTGGCGGCGATCGGAGGCACAAGCGCATGATCAAGAACAGAGAATCTGCAG
CTCGGTCCCGGGCTAGAAAGCAGGCTTACACAGTTGAGTTGGAACGTGAAGCTGCTCATTTAGCACAAGAGAATG
CCAAGCTTAGAAGGCAGCAAGAAAGGTTCTTGGCAGCAGCTCCTGCTCAGCTACCAAAAAAAAAAACCCTCTATA
GAACCTCAACAGCTCCATTTTGAgaatacattaatcccacttgctctattcacccatctttctggcttcccaaatttatccttaaaggagagtga
gatgtttccaatgtaatattgcaatgggcttttgctgttgtgttagatacatctcaatcaagcggctgtggtgacaggaggacaaacaaaagctcggaa
agtggtaaggacaaaataaagttgcatggattggaagccgcaggaccagtggagggttcttattttgtttgctttaactattcctcgactctcttgtctt
ctttcccatttctgcttttatgcttcttcccactaaagacgctgggatgttgggacagataaatgcgagtgaaatagcattttctagtctttttgcctgtaaa
tatgcatctccgtttg

>*PdFDL2.1*
cagaaagaaacatcatttcatctccagttactcctagtgctgttttccctgtcaaagcATGTGGTCATCGCCAGGAGCAAATATTGATAA
CAACAACACGAGCAACAGTAAAGTCTCTGGCAATTCTCCTTCAAAATGCTTTTCCTCTACATGTTCTTCTCCTTCACC
TTTCTCTCCTTCTCCTCCAATACCAAACCAATCAATGAACGGAGCCTCAATGGAAGAAGTTTGGGATGACATAAACC
TAGCTTCTCTTCATGATCATTCAAATACTAACACAAGCAGCAACACCAACCACCATTCTTTTAATGGTATGGTCTTTC
AAGATTTCTTGGCTAGACCTTCCAATAAAGACACATCAACAAGGGCTGCCTCTAAGGAACCCTCCTCTGGCGGGGG
TAACAGTTTCTTGAAGAAATCTTTAGGGCCACCCCCAGCTACCATGCTGAGTTTGAATTCTGGGTCTGATCATTTTC
ATTATCTGGAAAGCAGTGATACTGTCCCTGTGAGGCCAAATCCGCAAATGCATAGTCATGCCAATGGTGGCACAAT
AAGTTTTGATTCTTCTCTCGATTCCCCCTTTGATGCCTTGGGATCTTCTTCAGCGTTCCTTTCCATTTGCAAAAAAAG
GCCTCAAGAAAACGGTGATGTCTCTGGCGGCGATCGGAGGCACAAGCGCATGATCAAGAACAGAGAATCTGCAG
CTCGGTCCCGGGCTAGAAAGCAGGAATCTAGCTCTCCTTTTGAAAATTTGTTTTTAGTGAAATTTAATGATTATAGA

ATGTTAATGTTTTATCTTTTACTAATTTTGCAGGCTTACACAGTTGAGTTGGAACGTGAAGCTGCTCATTTAGCACA
AGAGAATGCCAAGCTTAGAAGGCAGCAAGAAAGGTTCTTGGCAGCAGCTCCTGCTCAGCTACCAAAAAAAAAAAC
CCTCTATAGAACCTCAACAGCTCCATTTTGAgaatacattaatcccacttgctctattcacccatctttctggcttcccaaatttatccttaa
aggagagtgagatgtttccaatgtaatattgcaatgggcttttgctgttgtgttagatacatctcaatcaagcggctgtggtgacaggaggacaaacaa
aagctcggaaagtggtaaggacaaaataaagttgcatggattggaagccgcaggaccagtggagggttcttatttttgtttgctttaactattcctcga
ctctcttgtcttctttcccatttctgcttttatgcttcttcccactaaagacgctgggatgttgggacagataaatgcgagtgaaatagcattttctagtcttt
ttgcctgtaaatatgcatctccgtttg

>*PdFDL3*
ctttgttggcatccaatgttttgtgccgtttaactttttgtgtgaacatttcagttgctttgctatctagctaagcgactcttccaaATGTTGTCGCCAA
CAGATTGCGAAGGCACCAGCTACAACAATAAGACAAAGAGTCTCCGAAAAGTCTCATCATCAATATCTTCAAAATC
ATCCTCCACTTCCTCATCTCCTTCAATATTTTCACCCTCCAATAATCTTCACTGCCAAGCCCAACAGAAGGCCGAAAC
CATGGAAGAAGTGTGGAAAGACATAAGTCTTGCTTCTCTTCATGACCACACTTCTACCGATCAAGAACTTTCCATG
ACCCCAAGACTTCACAACATTTCTCATCATCACCGTCATCATCACCATCATCATAATAATAACGGCCCTAACTTTATC
CTTCAAGATTTTCTTGCTAGGCCTTTCAACAAAGACCCACCAACAAGAATGGTCTCTATTATTCGAGACAGCACTCC
TTTTGGCTCTTCAGCGCCACCTCCTGCTACTGTTTCGAGTTTGAACTCTGGCCCTGGCTTTGATTTTCTTGAGAACTC
TGATCATCCTCAGAGGCCTGACTCGCAATTACAGAGCAACCCAATTTCAAATATTTCCTCATTCACTAGTCCTTTTGA
GGGTTTAGATTCATCTCCCGGCTTGCCTTCTTTTTGTAAGAAAAGAACTCAAGAATCTGATGGTAGTTCTGGTGATC
GCAGACACAAAAGAATGATCAAGAATCGAGAATCTGCAGCTCGTTCTAGAGCTAGAAAACAGGCTTACACAAACG
AATTGGAGAATGAAGTTGAACAGTTATTGAAAGAAAATGCAAGACTCAAGAGACAACAAGAAGAGCTATATTTG
GCCGCAGCAGCTCAACTACCAAAAAAGCACACCCTACAAAGAACATCAACAGCTCCATTTTGAgaactggtgtagatgta
tcagtgcttttagtttttaccttgtgcttctttaccctaataccacagtactatctccctattggggcaggagggggaaaagcattgatgtttttgcaagaa
agggtgtagctctagcaaatactgttgttttaagaaagaacaagtgtctaggaggtaaccgatgttgtttctggcattccaaacaggtaggagcttaat
ttggtgggtcttagttttggctttgtattctcttgtcctgcaaacatggggttgaaggggtaaattagggaaacaatattgtgatgttggactcagcccca
gcgagttgtacagtgaaaaatgatgggacattttgttctgaaactaagcaatgagcccatcaatttgaactaaaaaaaaaaaaaaaaaaaaaaa
aaaa

Supplementary Figure S1. Relationships of *Populus FD* homologs. (A) Maximum-likelihood phylogenetic analysis was performed on a protein sequence alignment. Bootstrap values >50% are shown at nodes. *P. deltoides* proteins are in blue type and Arabidopsis are in green type. See Table S2 for a complete list of sequence IDs, species and database sources. (B) Summary of alternate names and differences among cloned and predicted transcripts. (C) Alignment of *P. deltoides* and Arabidopsis FD family proteins. *P. deltoides* proteins were predicted from cDNAs cloned in this study. Motifs identified by Tsuji et al. (2013) are labeled and highlighted in different colors. (D) Sequences of *P. deltoides* cDNAs isolated in this study.

A

```
aLAP1b                  GCAATTAAAGAGCAAAATAACATGTTAGTGAAGCAGATCAAGGAGAAGGAG---------
Potri.008G098500.1      GCAATTAAAGAGCAAAATAACATGTTAGTGAAGCAGATCAAGGAGAAGGAG---------
Podel.08G116200.1       GCAATTAAAGAGCAAAATAACATGTTAGTGAAGCAGATCAAGGAGAAGGAG---------
Podel.08G117500.1       GCAATTAAAGAGCAAAATAACATGTTAGTGAAGCAGATCAAGGAGAAGGAG---------
aLAP1a                  GCAATTCAAGTGCAAAACAACATGTTAGTGAAGCAGATCAAGGAGAAGGAGAAGAAGGAT
Potri.010G154100.1      GCAATTCAAGTGCAAAACAACATGTTAGTGAAGCAGATCAAGGAGAAGGAGAAGAATGAT
Podel.10G155600.1       GCAATTCAAGTGCAAAACAACATGTTAGTGAAGCAGATCAAGGAGAAGGAGAAGAATGAT
aFUL                    GCGCTCCAGGAGCAAAACAACATGCTTGCAAAGAAGGTCAAGGAAAAGGAG---------
Potri.012G062300.1      GCGCTCCAGGAGCAAAACAACATGCTTGCAAAGAAGGTCAAGGAAAAGGAG---------
Podel.12G064600.1       GCGCTCCAGGAGCAAAACAACATGCTTGCGAAGAAGGTCAAGGAAAAGGAG---------
aMADS28                 GAACTGCAGGAGCAGAACAACATACTGGCAGAACAGGTAAAGGAAAATGAG---------
Potri.004G115400.1      GAACTGCAGGAGCAGAACAACATACTGGCAGAACAGGTAAAGGAAAATGAG---------
Podel.T190600.1         GAACTGCAGGAGCAGAACAACATACTGGCAGAACAGGTAAAGGAAAATGAG---------
aMADS14                 GCACTTCAGGACCAGAACAACATATTAGCAGAACAGGTCAAGAAAAAACTG---------
Potri.017G099800.1      GCACTTCAGGACCAGAACAACATACTAGTAGAACAGGTCAAGAAAAAACTG---------
Podel.17G102400.1       GCACTTCAGGACCAGAACAACATACTAGTAGAACAGGTCAAGAAAAAACTG---------
Podel.17G101400.1       GCACTTCAGGACCAGAACAACATACTAGTAGAACAGGTCAAGAAAAAACTG---------
                        *   *  * *  ** ** *****  * *   *  ** * *** * **   *

aLAP1b                  AAAGCACTGG---CACAGCCGGCACTCTGGGATCAGCAAGATCATGGCCCTAATGCTTCC
Potri.008G098500.1      AAAGCACTGG---CACAGCCGGCACTCTGGGATCAGCAAGATCATGGCCCTAATGCTTCC
Podel.08G116200.1       AAAGCACTGG---CACAGGCGGCACTCTGGGATCAGCAAGATCATGGCCCTAATGCTTCC
Podel.08G117500.1       AAAGCACTGG---CACAGGCGGCACTCTGGGATCAGCAAGATCATGGCCCTAATGCTTCC
aLAP1a                  AAAGCTGTGG---CACAGCCGGCATTCTGGGATCAGCAAAATCACGGCCCTGATGCTTCC
Potri.010G154100.1      AAAGCGCTGG---CACAGCCGGCATTCTGGGATCTGCAAGATCATGGCCCTAATGCTTCC
Podel.10G155600.1       AAAGCGCTGG---CACAGCCGGCATTCTGGGATCTGCAAAATCATGGCCCTAATGCTTCC
aFUL                    AAGGCAATAATAGCTCAACAAGCACCATGGGAGCAGCAAAATCCTGACCTGGATTCATCT
Potri.012G062300.1      AAGGCAATAATAGCTCAACAAGCATCATGGGAGCAGCAAAATCCTGACCTGGATTCGCCT
Podel.12G064600.1       AAGGCAATAATAGCTCAACAAGCATCATGGGAGCAGCAAAATCCTGACCTGGATTCGCCT
aMADS28                 AAGTCACTGA---CTGAGCAAGCTCAATGGGAGCAGCGAAACCTTGGCCAAAGCTCATCC
Potri.004G115400.1      AAGTCACTGA---CTGAACAAGCTCAATGGGAGCAGCGAAACCTTGGCCAAAACTCATCC
Podel.T190600.1         AAGTCGCTGA---CTGAGCAAGCTCAATGGGAGCAGCGAAACCTCGGTCAAAACTCATCC
aMADS14                 AAGGCGCTAA---CTGAGCAAGCACAATGGGAGCAGCAAAACCTTGGCCAGAACTCATCC
Potri.017G099800.1      AAGGCGCTAA---CCGAGCAAGCACAATGGGAGCAGCAAAACCTTGGCCAGAACTCATCC
Podel.17G102400.1       AAGGCGCTAA---CTGAGCAAGCACAATGGGAGCAGCAAAACCTTGGCCAGAACTCATCC
Podel.17G101400.1       AAGGCGCTAA---CTGAGCAAGCACAATGGGAGCAGCAAAACCTTGGCCAGAACTCATCC
                        **  *  *     *  *    **    ***** * ** * * *  *  *      *  *

aLAP1b                  TCATTCCTTTTACCACAA---------------------------CCGCCACTGCCTTGT
Potri.008G098500.1      TCATTCCTTTTACCA------------------CAGCT---------GCCACTGCCTTGT
Podel.08G116200.1       TCATTCCTTTTACCA------------------CAGCC---------GCCACTGCCTTGT
Podel.08G117500.1       TCATTCCTTTTACCA------------------CAGCC---------GCCACTGCCTTGT
aLAP1a                  TCATTCCTTTTATCA------------------CAGCCGGCAGGCCTGCCACTGCCTTGC
Potri.010G154100.1      TCATTCCTTTTATCA------------------CAGCCGGCAGGCCTGCCACTGCCTTGC
Podel.10G155600.1       TCATTCCTTTTATCA------------------CAGCCGGCAGGCCTGCCACTGCCTTGC
aFUL                    ACAATTCTTCGGCCACAA---------CCAATGCAG---------------------CCC
Potri.012G062300.1      ACAATTCTTCGGCCACAA---------CCAATGCAG---------------------CCC
Podel.12G064600.1       ACAATTCTTCGGCCACAA---------CCACTGCAG---------------------CCC
aMADS28                 TCTTTTATGCCACCAGTAGTACAACCTCCGCTGCAGCCACCAATGTCACCACATCCTCCT
Potri.004G115400.1      TCTTTTATGCCACCGGTAGTACAACCTCCGCTGCAGCCACCAATGCCACCACATGCTCCT
Podel.T190600.1         TCTTTTATGCCACCGGTAGTACAACCTCCGCTGCAGCCACCAATGCCACCACATGCTCCT
aMADS14                 TCTTTTATGCTACCACAAGCACAACCTCCACTGCAGCCGTCGATGCTATCACTTCCTCCA
Potri.017G099800.1      TCTTTTATGCTACCACAAGCACAACCTCCACTGCAGCCGTCGATGCTATCACATCCTCCA
Podel.17G102400.1       TCTTTTATGCTGCCACAAGCACAACCTCCACTGAAGCCGTCGATGCTATCACATCCTCCA
Podel.17G101400.1       TCTTTTATGCTACCACAAGCACAACCTCCACTGCAGCCGTCGATGCTATCACATCCTCCA
                         *  *  *     *

aLAP1b                  TTAAACAT------CAGTTACCAGG-AAGAAGATCCA------ATAGCGAGGAGGA---A
Potri.008G098500.1      TTAAACAT------CAGTTACCAGG-AAGAAGATCCA------GAAGCGAGGAGGAATTA
Podel.08G116200.1       TTAAACAT------CAGTTACCAGG-AAGAAGATCCA------GAAGCGAGGAGGAATTA
Podel.08G117500.1       TTAAACAT------CAGTTACCAGG-AAGAAGATCCA------GAAGCGAGGAGGAATTA
aLAP1a                  TTAAACATCGGTGGCAGTTACCAGG-AAGAAGCTCCT------GAAGCGAGGAGGA---A
```

```
Potri.010G154100.1     TTAAACATCGGTGGCAGTCACCAGG-AAGAAGCTCCT------GAAGCGAGGAGGA---A
Podel.10G155600.1      TTAAACATCGGTGGCAGTCACCAGG-AAGAAGCTCCT------GAAGCGAGGAGGA---A
aFUL                   TTAAACATAAGTAGCAGCCACCTGGCAACGGGA------------AATGAGGAAGA---A
Potri.012G062300.1     TTAAACATAAGTAGCAGCCACCTGGCAACGGGA------------ATTGAGGAAGA---A
Podel.12G064600.1      TTAAACATAAGTAGCAGCCACCTGGCAACGGGA------------ATTGAGGAAGA---A
aMADS28                TTAACTATAGGTGACAGTTTCCAGATTATAGGATTCCTCAACGGAAATGAGAATGT---T
Potri.004G115400.1     TTAACTATAGGTGACAGTTTCCAGATTATAGGATTCCTCAACGGAAATGAGAATGT---T
Podel.T190600.1        TTGACTATAGGTGACAGTTTCCAGATTATAGGATTCCTCAACGGAAATGAGAATGT---T
aMADS14                CCAACTATAGGTGGCAGTTTCCAGATTAGAGGATTCCTGAACGGAAACAAGGATGT---T
Potri.017G099800.1     CCAACTATAGGTGGCAGTTTCCAGATTAGAGGATTCCTGAACGGTAACAAGGATGT---T
Podel.17G102400.1      CCAACTCTAGGTGGCAGTTTCCAGATTAGAGGATTCCTGAACGGAAACAAGGATGT---T
Podel.17G101400.1      CCAACTATAGGTGGCAGTTTCCAGATTAGAGGATTCCTGAACGGAAACAAGGATGT---T
                          *   *      ***   ** *   *   *             *   ** * *

aLAP1b                 TGAACTTGACCTCACGCTGGAACCAATTTATTCGTGTCACCTTGGATGCTTTGGAACATG
Potri.008G098500.1     TGAACTTGACCTCACGCTGGAACCAATTTATTCGTGTCACCTTGGATGCTTTGGAACATG
Podel.08G116200.1      TAAACTTGACCTTACGCTGGAACCAATTTATTCGTGTCACCTTGGATGCTTTGGAACGTG
Podel.08G117500.1      TAAACTTGACCTTACGCTGGAACCAATTTATTCGTGTCACCTTGGATGCTTTGGAACGTG
aLAP1a                 TGGGCTTGGCCATACGCTGGAACCAATTTATTCTTTTCACCTTGGAGGTTACGGAGCATG
Potri.010G154100.1     TGAGCTTGGCCATACGCTGGAACCAATTTATTCTTTTCACCTTGGAGGTTACGGAGCATG
Podel.10G155600.1      TGAGCTTGGCCATACGCTGGAACCAATTTATTCTTTTCACCTTGGAGGTTACAGAGCATG
aFUL                   CCCACTCCAATCCAACATCGAGCCAACGCACTGTTGCCTGCTTGGATGCTTCGCTACCTC
Potri.012G062300.1     CCCGCTCCAATCCAACATCGAGCCAACGCACTGTTGCCTGCTTGGATGCTTCGCTACCTC
Podel.12G064600.1      CCCACTCCAATCCAACATCGAGCCAACGCACTGTTGCCTGCGTGGATGCTTCGCTACCTC
aMADS28                GAGGTTCAAACTCCACCTAGCACCA---------TGCCATCTTGGATGCTTCGCCATGTC
Potri.004G115400.1     GAGGTTCAAACTCCACCTAGCACCA---------TGCCATCTTGGATGCTTCGCCATGTC
Podel.T190600.1        GAGGTTCAAACTCCACCTAGCACCA---------TGCCATCTTGGATGCTTCGCCATGTC
aMADS14                GAAGTTCAAACTCAACCTAGCACCA---------TGCCACATTGGATGCTTCGCCATGTC
Potri.017G099800.1     GAAGTTCAAACTCAACCTAGCACCA---------TGCCACATTGGATGCTTCGCCATGTC
Podel.17G102400.1      GAAGTTCAAACTCAACCTAGCACCA---------TGCCACATTGGATGCTTCGCCATGTC
Podel.17G101400.1      GAAGTTCAAACTCAACCTAGCACCA---------TGCCACATTGGATGCTTCGCCATGTC
                            *           * *  ***         *  *    **** * *        *

aLAP1b                 AATA-----------------------------------TTTGGGTGAAGAAAGAC----
Potri.008G098500.1     AATA-----------------------------------TTTGGGTGAAGAAAGAC----
Podel.08G116200.1      AATA-----------------------------------TTTGGGTGAAGAAAGAC----
Podel.08G117500.1      AATA-----------------------------------TTTGGGTGAAGAAAGAC----
aLAP1a                 AATA-----------------------------------TTTGGGTGAAGAAAGAT----
Potri.010G154100.1     AATA-----------------------------------TTTGGGTGAAGAAAGAT----
Podel.10G155600.1      AATA-----------------------------------TTTGGGTGAAGAAAGAT----
aFUL                   AACGAATAAAAGAATCAAATCAACTCCTTGTTGCGCTA-TTTGTATACACCAAGAC----
Potri.012G062300.1     AACGAATAAAAGAATCAAATCAACTCCTTGTTGCGCTGTTTTGTATACACCAAGAC----
Podel.12G064600.1      AACGAATAAAAGAATCAAATCAACTCCTTGTTGCGCTGTTTTGTATACACCAAGAC----
aMADS28                AACG-ATACAA----------------------------TTTG---------AGAC----
Potri.004G115400.1     AACG-ATACAA----------------------------TTTG---------AGAC----
Podel.T190600.1        AACG-ATACAA----------------------------TTTG---------AGAC----
aMADS14                ACTG-ATAGAA----------------------------TTTG---------AGACATGG
Potri.017G099800.1     AATG-ATAGAA----------------------------TTTG---------AGACACGG
Podel.17G102400.1      AATG-ATAGAA----------------------------TTTG---------AGACATGG
Podel.17G101400.1      AATG-ATAGAA----------------------------TTTG---------AGACATGG
                       *                                      ****         ***

aLAP1b                 GCTCTGAAACTG----CTAAAAATGTAGTTTTTGATTCGTGATGAAAATAGAT------T
Potri.008G098500.1     ACTCTGAAACTG----CTAAAAATGTAGTTTTTGGTTCGTTATGAAATTAGATTATATAT
Podel.08G116200.1      ACTCTGAAACTG----CTAAAAATGTAGTTTTTGGTTCGTTATGAAATTAGAT------T
Podel.08G117500.1      ACTCTGAAACTG----CTAAAAATGTAGTTTTTGGTTCGTTATGAAATTAGAT------T
aLAP1a                 -ACTCGAAATTAAGCACTTATAATGAAGTTTTGA------TATTAAAATATAT----ATT
Potri.010G154100.1     ACTCGAA-----AGCACTAATAATGAAGTTATGA------TATTAAAATATAT----ATT
Podel.10G155600.1      ACTGGAA-----AGCACTAATAATGAAGTTTTGA------TATTAAAATATAT----ATT
aFUL                   GTGGGGGTATGGAAATGTA--AATCCATTTTCTGTCTGATGAAGCGAAAATGTTGGATCG
Potri.012G062300.1     GTGGGGGTATGGAAATGTA--AAT-CATTTTCAGTCTGATGAAGTGAAAATGTTGGATCG
Podel.12G064600.1      GTGGGGGTATGCAAATGTA--AATCCATTTTCAGTCTGATGAAGTGAAAATGTTGGATCG
aMADS28                -TTGGGAGATCGGAAAATA--AATGGGTTATTGAAGTGAAGAAGAACA---------TTT
```

Potri.004G115400.1 -ATGGGAGATCGGAAAATA--AATGGGTTATTGAAGTGAAGAAGAGCA---------TTT
Podel.T190600.1 -ATGGGAGATCGGAAAATA--AATGGGTTATTGAAGTGAAGAAGAGCA---------TTT
aMADS14 GATGGGAGATCAGAAAATC--AATGGGGCATTGAGGTGAAGAAGAAGA------GTATTT
Potri.017G099800.1 GATGGGAGATCAGAAAATC--AATGGGGCATTGAGGTGAAGAAGAGTA---------TTT
Podel.17G102400.1 GATGGGAGATCAGAAAATC--AATGGCGCGTTGAGGTGAAGAAGAGTA---------TTT
Podel.17G101400.1 GATGGGAGATCAGAAAATC--AATGACGCGTTGAGGTGAAGAAGAGTA---------TTT

aLAP1b AT------ATACACACATTGTTATAACTAAGGTTTCATGATCATGTCAAATTGAT-----
Potri.008G098500.1 *AT------ATACGCACCTTGTTATAACTAAGGTTTCATCATGT---CAAATTGAT-----*
Podel.08G116200.1 AT------ATACGCACCTTGTTATAACTAAGGTTTCATCATAT---CAAATTGAT-----
Podel.08G117500.1 AT------ATACGCACCTTGTTATAACTAAGGTTTCATCATAT---CAAATTGAT-----
aLAP1a AT------GTGTGTTGATGGTAATAACCAATGTATGTCG-------GATGTGAATTCGGA
Potri.010G154100.1 *AT------GTGTGTTGATGGTAATAACCAA--ATGTATGTCGG---AATGTGAATTCGGA*
Podel.10G155600.1 AT------GTGTGTTGATGGTAATAACCAA--ATGTATGTCGG---AATGTGAATTCTGA
aFUL GTTGCTTGATACTTGGGAATTATCTAGCAG--TTCCATGATAG---TA-ATGTATCT---
Potri.012G062300.1 GTTGCTTGATACTTGGGAATTATCTAGCAG--TTCCATGATAG---TA-ATGTATCT---
Podel.12G064600.1 GTTGCTTGATACTTG---ATTATCTAGCAG--TTCCATGATAG---TA-ATGTATCT---
aMADS28 ATCACTTCGTTCATT-----TATCCATCAA--TGTATTCATCA---CACA---ATCT---
Potri.004G115400.1 ATCCCTTCGTTCATA-----TAGCCATCAA--TGTATTCATCA---CACA---ATCT---
Podel.T190600.1 ATCACTTCGTTCACA-----TAGCCATCAA--TGTATTCATCA---CACA---ATCT---
aMADS14 ACCACTTCGTTCTTA-----TAGCCGTCAA--TATATCCATCG---CATA----------
Potri.017G099800.1 ATCACTTCGTTCTTA-----TAGCCGTCAA--TTTATCCATCG---CATAATTACCT---
Podel.17G102400.1 ATCACTTCGTTCTTA-----TAGCCGTCAA--TTTATCCATCG---CATAATTACCT---
Podel.17G101400.1 ATCACTTCGTTCTTA-----TAGCCGTCAA--TTTATCCATCG---CATAATTACCT---

aLAP1b ---------ATAATTAATGACATGATCCTTTGA--TCGTATATCAATTATCTA-------
Potri.008G098500.1 *---------ATAATTAATGACATGATCCTTTGA--TCGTATATCAATTATCCA*-------
Podel.08G116200.1 ---------ATAATTAATGACATGATCCTTTGA--TCGTATATCAATTATCTA-------
Podel.08G117500.1 ---------ATAATTAATGACATGATCCTTTGA--TCGTATATCAATTATCTA-------
aLAP1a TGTTTCCAGTTAATTATTATTGTTGTTGTTAAAAGTTGTATCTGAATCAATTAAGAAGAC
Potri.010G154100.1 *TGTTTCAAGTTAAGTGTTGTTGTTGTTGTTAAAAGTTGTATCTGAATCAATTAAGAAGAC*
Podel.10G155600.1 TGTTTCATGTTAATTGTTGTTGTTGTTGTTAAAAGTTGTATCTGAATCAATTAAGAAGAC
aFUL ---------CT-----ATGTATCTATAATTGTAA----TGTATCA---------CTA-AA
Potri.012G062300.1 ---------CT-----ATGTATCTATAATTGTAA----TGTATCA---------CTA-AA
Podel.12G064600.1 ---------CT-----ATGTATCTATAATTGTAA----TGTATCA---------CTA-AA
aMADS28 ---------CTACTAAATACGCTGACAATTACAAGTGCTT**GAGCA**---------**CAAGAA**
Potri.004G115400.1 ---------CTACTAAATATGCTGACAATTACAAGTGCTTGTTCA---------CAAGAA
Podel.T190600.1 ---------CTACTAAATATGCTGACAATTACAAGTGCTTGTTCA---------CAAGAA
aMADS14 ---------ACCTCTATTAATATGCTTA**CTTCAAGTGCTAAGTCA**---------**CAATAA**
Potri.017G099800.1 ---------CTACT-AATATGCTGACAATTTCAAGTGCTTATTCA---------CAATAA
Podel.17G102400.1 ---------CTACT-AATATGCTGACAATTTCAAGTGCTTCTTCA---------CAATAA
Podel.17G101400.1 ---------CTACT-AATATGCTGACAATTTCAAGTGCTTCTTCA---------CAATAA

aLAP1b TCTTGTTGGTAATAATTAAATTGTTGACGG-AATCTTAATTCCGATGTTTCAAGTTGTGT
Potri.008G098500.1 TCTTGTTGGTAATAATTAAATTGTTGATGG-AATCTTAATTCCGATGTTTCAAGTCGTGT
Podel.08G116200.1 TCTTGTTGGTAATAATTAAATTGTTGATGG-AATCTTAATTCC-----------------
Podel.08G117500.1 TCTTGTTGGTAATAATTAAATTGTTGATGG-AATCTTAATTCCGATGTTTCAAG------
aLAP1a TCCTACAGCTGATTTTCATAATAT--ATTGGAAGGTCAATTACTGT--------------
Potri.010G154100.1 TCCTACAGCTAATTTTCATAATTT--ATTGGAAGGTTAATTACTGTTAGTAGCCCCGTCA
Podel.10G155600.1 TCCTACAGCTGATTTTCATAATAT--ATTGGAAGGTTAATTACTGTTACTAGCCCCGTCA
aFUL TTGCTTGTGTAA--TAAAATCCATAAATAGAAAAA-------------------------
Potri.012G062300.1 TTGCTTGTGTAA--TAAAATCCATAAATAGAAAGACTAACTGCAATG---TACCATGGAC
Podel.12G064600.1 TTGCTTGTGTAA--TAAAATCCATAAATAGAAAGACTAACTGCAATG---TACCATGGAC
aMADS28 **TGTATTTCGTAACTTTC**ACTATTTCAAGACCAAGACAAGCTCCGGTGCCTTGTCATATCT
Potri.004G115400.1 TGTATTTCGTAACTTTCACTATTTCAAGACCAAGACCAGCTCCGGTGCCTTGTCATATCT
Podel.T190600.1 TGTATTTCGTAACTTTCACTATTTCAAGACCAAGACCAGCTCCGGTGCCTTGTCATATCT
aMADS14 TTGAATTTGTAATCTTAACTATTTCGAAAAAAAAA-------------------------
Potri.017G099800.1 TTGAATTTGTAATCTTAACTATTTCGAGACCAAAACCAGCTCC-GTGTCTTGTCATATCC
Podel.17G102400.1 TTGAATTTGTAATCTTAACTATTTCGAGACCAAAACCAGCTCC-GTGTCTTGTCATATCC
Podel.17G101400.1 TTGAATTTGTAATCTTAACTATTTCGAGACCAAAACCAGCTCC-GTGTCTTGTCATATCC

B

>aFUL
GCACTTAAGCACGTGAGATCAAGAAAGAATCAGTTGATGTATGAATCCATTTCAGAGCTACAAAAGAAGGACAAG
GCGCTCCAGGAGCAAAACAACATGCTTGCAAAGAAGGTCAAGGAAAAGGAGAAGGCAATAATAGCTCAACAAGC
ACCATGGGAGCAGCAAAATCCTGACCTGGATTCATCTACAATTCTTCGGCCACAACCAATGCAGCCCTTAAACATA
AGTAGCAGCCACCTGGCAACGGGAAATGAGGAAGAAC***CCACTCCAATCCAACATCGAG***CCAACGCACTGTTGCCT
GCTTGGATGCTTCGCTACCTCAACGAATAAAAGAATCAAATCAACTCCTTGTTGCGCTATTTGTATACACCAAGACG
TGGGGGTATGGAAATGTAAATCCATTTT**CTGTCTGATGAAGCGAAAATGTTG**GATCGGTTGCTTGATACTTGGGA
ATTATCTAGCAGTTCCATGATAGTAATGTATCTCTATGTATCTATAATTGTAATGTATCACTAAATTGCTTGTGTAAT
AAAATCCATAAATAGAAAAAAAAAAAAAAAAA

>aMADS28
GCTCTTAAGCGCATACGATCAAGAAAGAACCAACTCATCCATGAATCCCTTAATGAGCTGCGGAAAAAAGAAAAG
GAACTGCAGGAGCAGAACAACATACTGGCAGAACAGGTAAAGGAAAATGAGAAGTCACTGACTGAGCAAGCTCA
ATGGGAGCAGCGAAACCTTGGCCAAAGCTCATCCTCTTTTATGCCACCAGTAGTACAACCTCCGCTGCAGCCACCA
ATGTCACCACATCCTCCTTTAACTATAGGTGACAGTTTCCAGATTATAGGATTCCTCAACGGAAATGAGAATGTTGA
GGTTCAAACTCCACCTAGCACCATGCCATCTTGG***ATGCTTCGCCATGTCAACG***ATACAATTTGAGACTTGGGAGAT
CGGAAAATAAATGGGTTATTGAAGTGAAGAAGAACATTTATCACTTCGTTCATTTATCCATCAATGTATTCATCACA
CAATCTCTACTAAATACGCTGACAATTACAAGTGCTT**GAGCACAAGAATGTATTTCGTAACTTTC**ACTATTTCAAGA
CCAAGACAAGCTCCGGTGCCTTGTCATATCTGCAAAAAAAAAAAAAAAAAAA

>aMADS14
GAACTTCAACACCTGGAGCAAAAAATAGATACTGCTCTTAAGAGCGTACGATCAAGAAAGAACCAACTCGTCCAT
GAATCCCTTGCAGAAATGCGGGAAAAAGAAAAGGCACTTCAGGACCAGAACAACATATTAGCAGAACAGGTCAA
GAAAAAACTGAAGGCGCTAACTGAGCAAGCACAATGGGAGCAGCAAAACCTTGGCCAGAACTCATCCTCTTTTAT
GCTACCACAAGCACAACCTCCACTGCAGCCGTCGATGCTATCACTTCCTCCACCAACTATAGGTGGCAGTTTCCAGA
TTAGAGGATTCCTGAACGGAAACAAGGATGTTGAAGTTCAAACTCAACCTAGCACCATGCCACATT***GGATGCTTCG***
CCATGTCACTGATAGAATTTGAGACATGGGATGGGAGATCAGAAAATCAATGGGGCATTGAGGTGAAGAAGAAG
AGTATTTACCACTTCGTTCTTATAGCCGTCAATATATCCATCGCATAACCTCTATTAATATGCTTA**CTTCAAGTGCTA**
AGTCACAATAATTGAATTTGTAATCTTAACTATTTCGAAAAAAAAAAAAAAAAA

Supplementary Figure S2. Alignment of 3' regions of *Populus AP1/FUL* family members. (A) *P. trichocarpa* and *P. deltoides* sequences are provided directly below their orthologous aspen sequences (*aLAP1a*, *aLAP1b, aFUL, aMADS14* and *aMADS28*). Stop codons are in red boxes and *P. trichocarpa in situ* probe sequences are shown in blue italic type. Forward primers for qRT-PCR are shown in bold, italic type and underlined. Reverse primers are in bold type and underlined. (B) Sequences of *aFUL, aMADS14* and *aMADS28* 3' RACE clones isolated in this study.

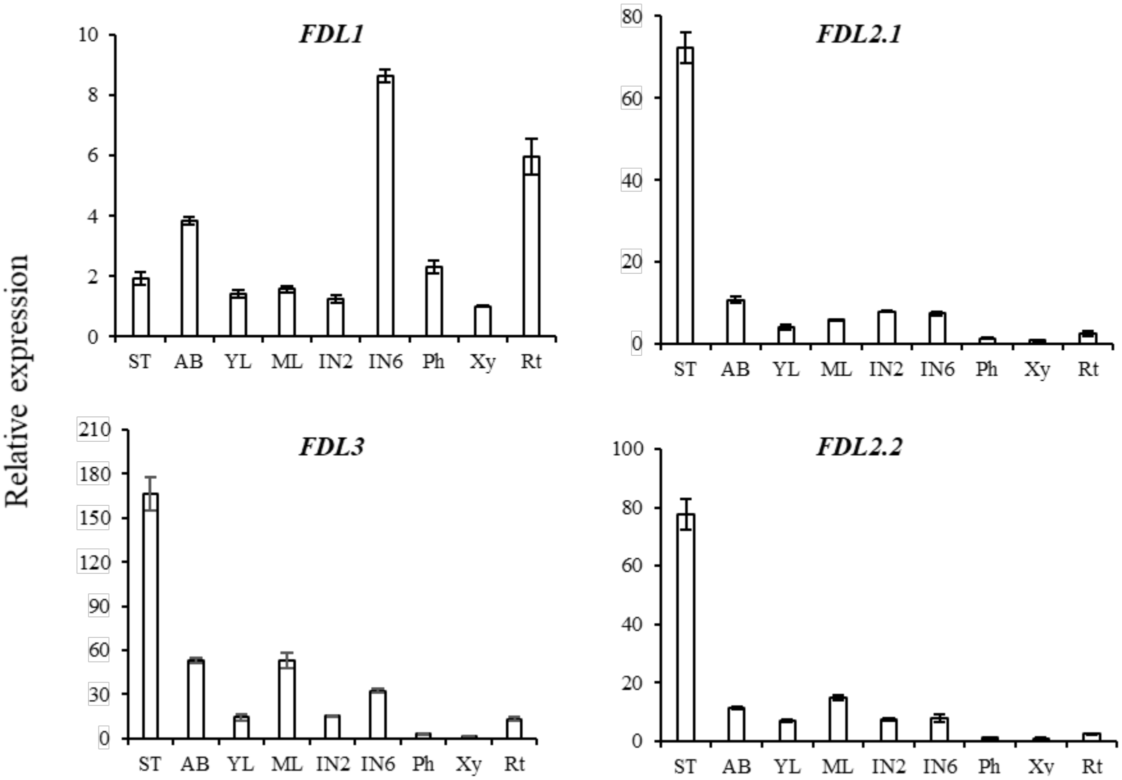

Supplementary Figure S3. Expression of *FDL* genes in various tissues of 4-month-old *P. tremula* × *P. alba* clone 717-1B4 trees. ST, shoot apices; AB, axillary buds; YL, young leaf (leaf plastochron index 2, LPI2. (LPI1: the first leaf from the top, whose lamina length is larger or equal of 1 cm); ML, nearly mature leaf (LPI6); IN2, internode beneath LPI2 (primary growth zone); IN6, internode beneath LPI6 (transitional zone from primary to secondary growth; Ph, phloem; Xy, xylem; Rt, root. All values were normalized against *UBQ2* and expressed as fold upregulation to the sample with the lowest expression. Error bars are ± SDs for three replicate reactions performed on a pooled sample from three one-meter high trees.

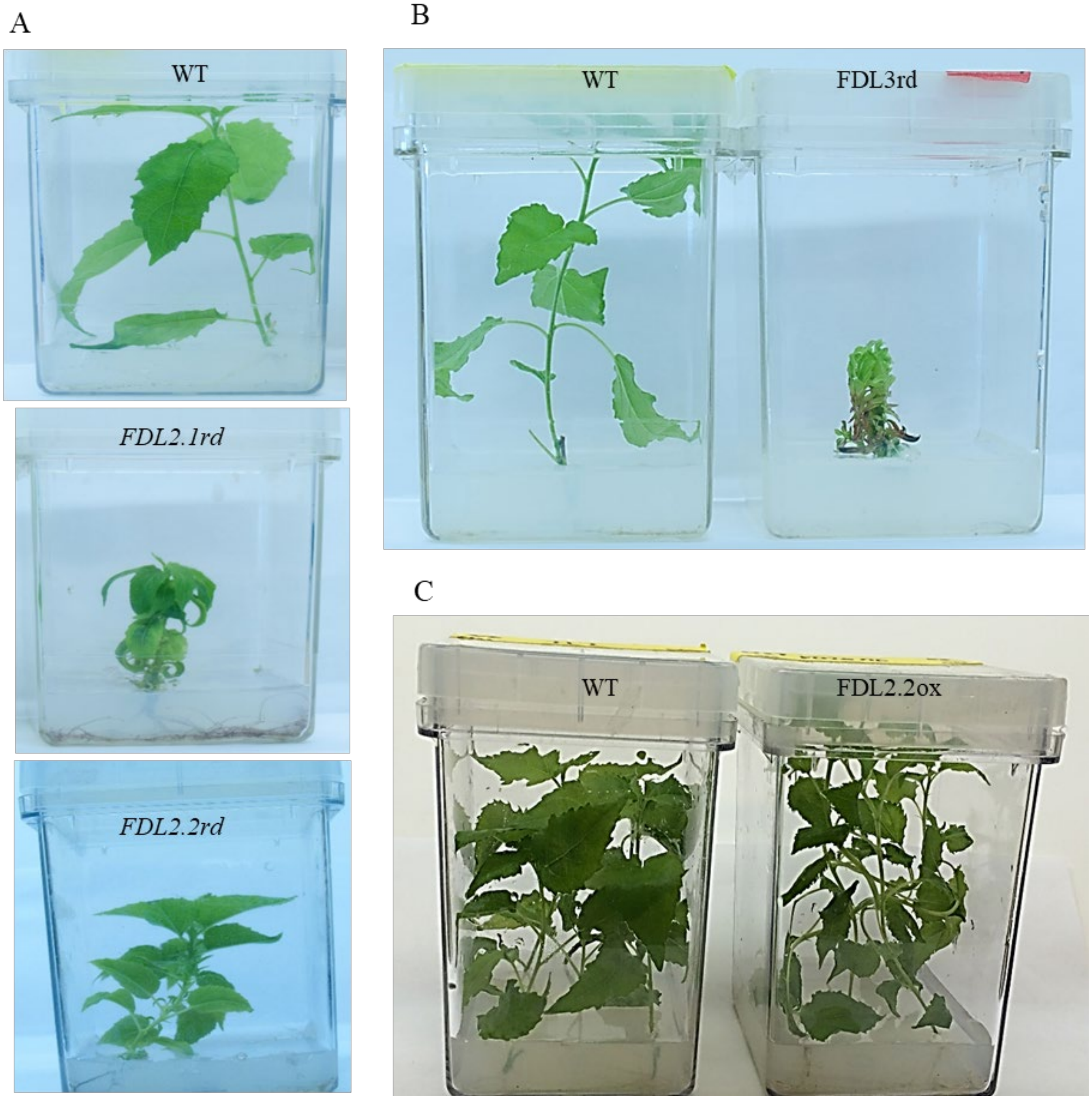

Supplementary Figure S4. Representative *in vitro* phenotypes of *FDL2.1*, *FDL2.2*, and *FDL3* transgenics. (A) Two-month old WT, *FDL2.1rd* and *FDL2.2rd* plants propagated at the same time. (B) Two-month old WT and a two-month old *FDL3rd* plant that did not root (C) Two-month-old WT and *FDL2.2ox* plants.

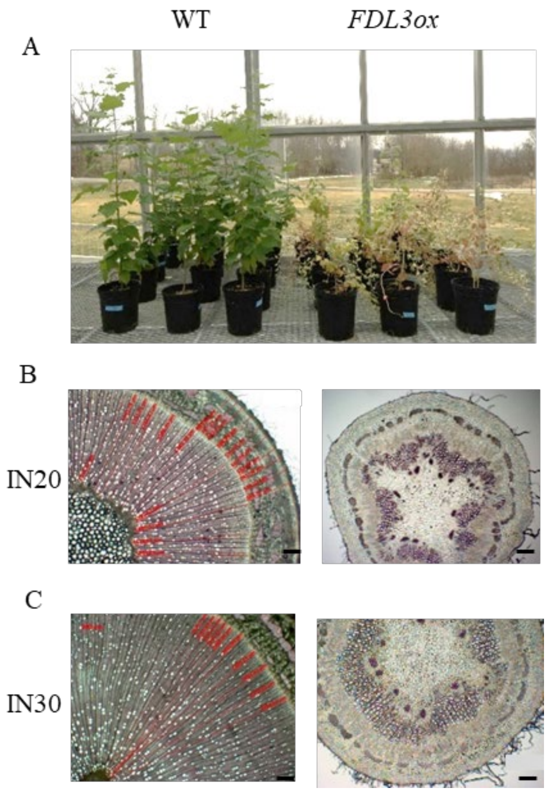

Supplementary Figure S5. *FDL3ox* plants show underdeveloped secondary growth. (A) Plants were grown under long daylength (LD) conditions in a greenhouse for 6 months, WT (left) compared with *FDL3ox* (right). (B-C) Transverse sections (60 µm thick) were from the 20th and 30th internodes (IN20, B and IN30, C). Scale bars = 200 µm.

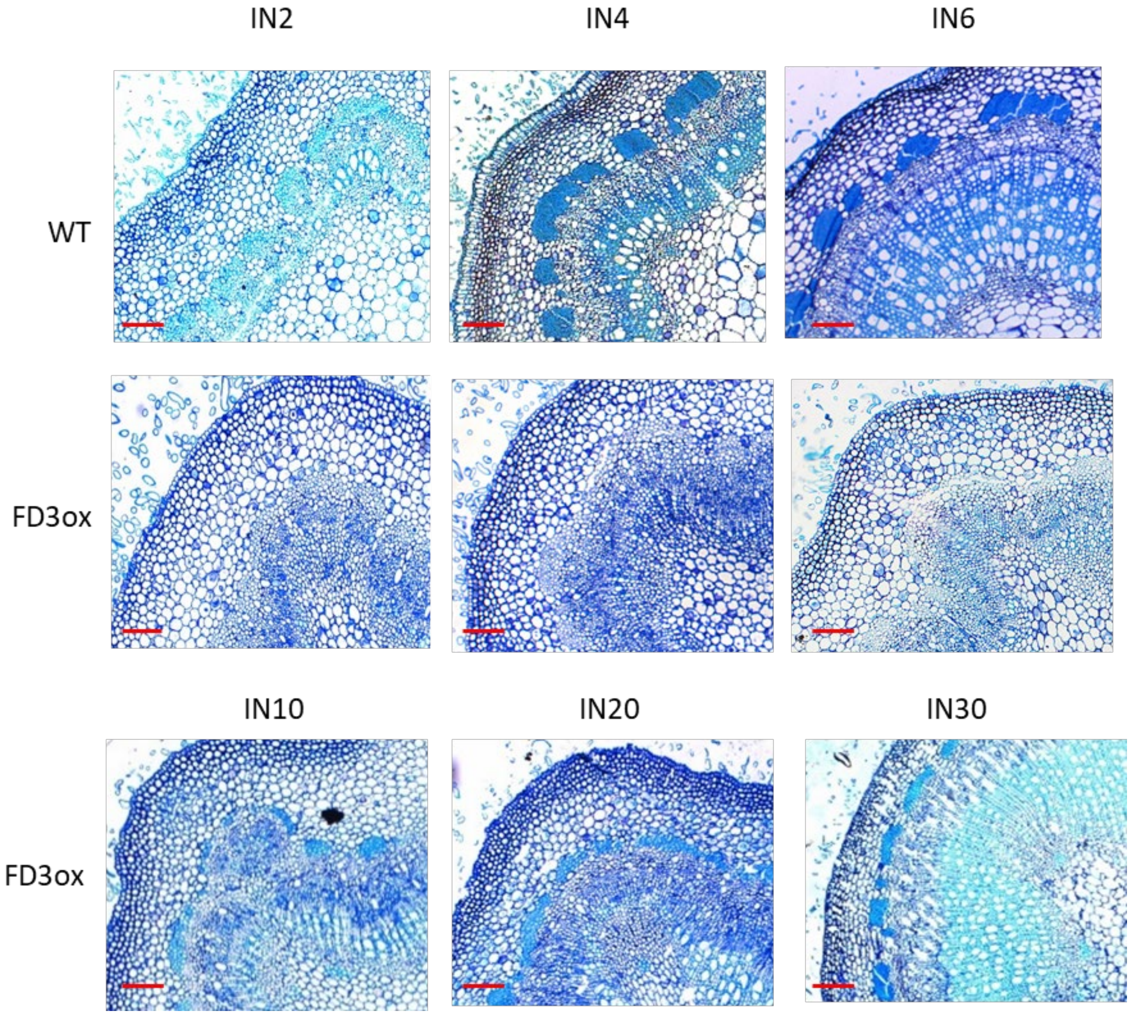

Supplementary Figure S6. Micrographs comparing secondary growth transitions in WT and *FDL3ox* transgenic. Trees were grown in a LD greenhouse. Sections (2 µm thick) were stained with toluidine blue. Scale bars = 100 µm**.**

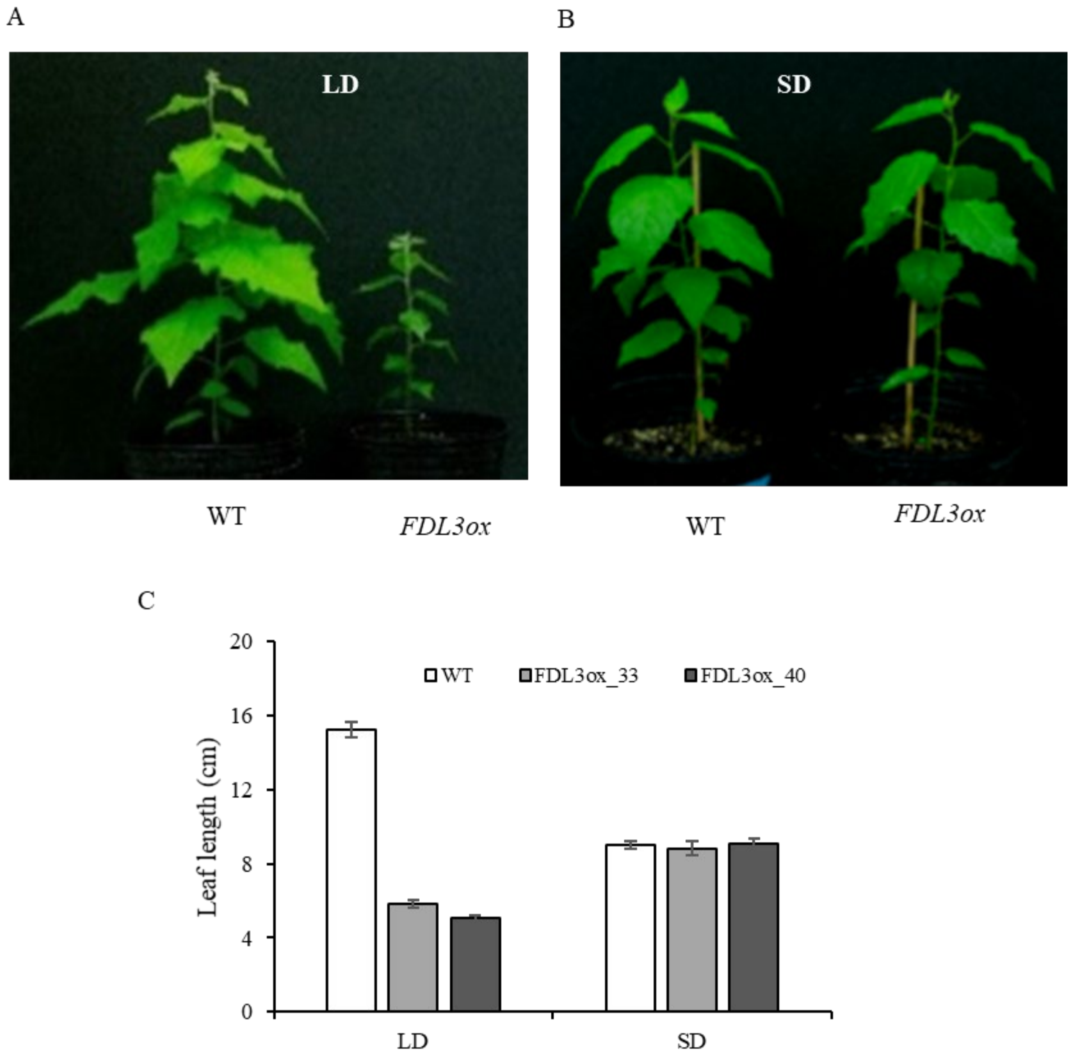

Supplementary Figure S7. Effect of short days on leaf size and height growth in young *FDL3ox* plants grown only in growth chambers. (A-B) After 2 weeks acclimation in soil in LDs, *FDL3ox* and WT plants were grown for 4 weeks in a LD growth chamber (A), or in a SD growth chamber (B). In contrast to significantly stunted growth of *FDL3ox* with small leaves compared with WT in LD, *FDL3ox* plants grew as WT in SD. (C) Leaf length of *FDL3ox* and WT plants in the LDs and SDs. Six fully expanded leaves were measured for each plant at the end of 4 weeks in LDs and SDs. Leaf lengths are means ± SE (n =4).

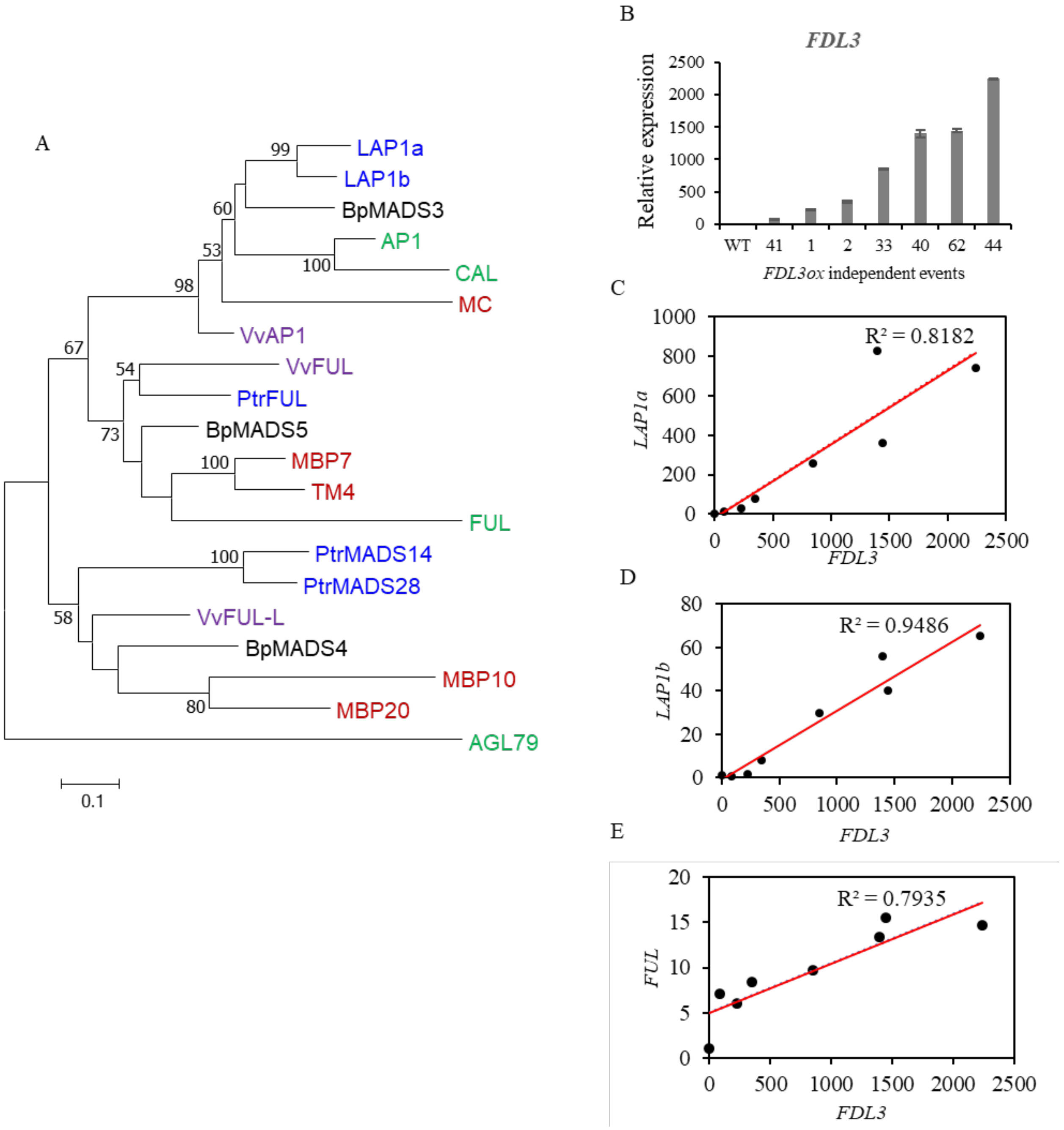

Supplementary Figure S8. Upregulation of three *AP1/FUL* family members is positively correlated with *FDL3ox* expression level. (A) Maximum likelihood phylogeny of AP1/FUL family proteins. Bootstrap values >50% are shown at nodes. Proteins from same species are in same color type: *P. trichocarpa* (blue), Arabidopsis (green), *Vitis vinifera* (purple), *Solanum lycopersicum* (red) and *Betula pendula* (black). See Supplementary Table S2 for a complete list of sequence IDs, species and source database. (B) *FDL3* expression in the shoot tips of WT and seven independent *FDL3ox* events of *in vitro* plants. Expression levels of (C) *LAP1a*, (D) *LAP1b*, and (E) *FUL* were correlated with that of *FDL3*. Expression of *MADS14* and *MADS28* was not detectable in WT or *FDL3ox* plants.

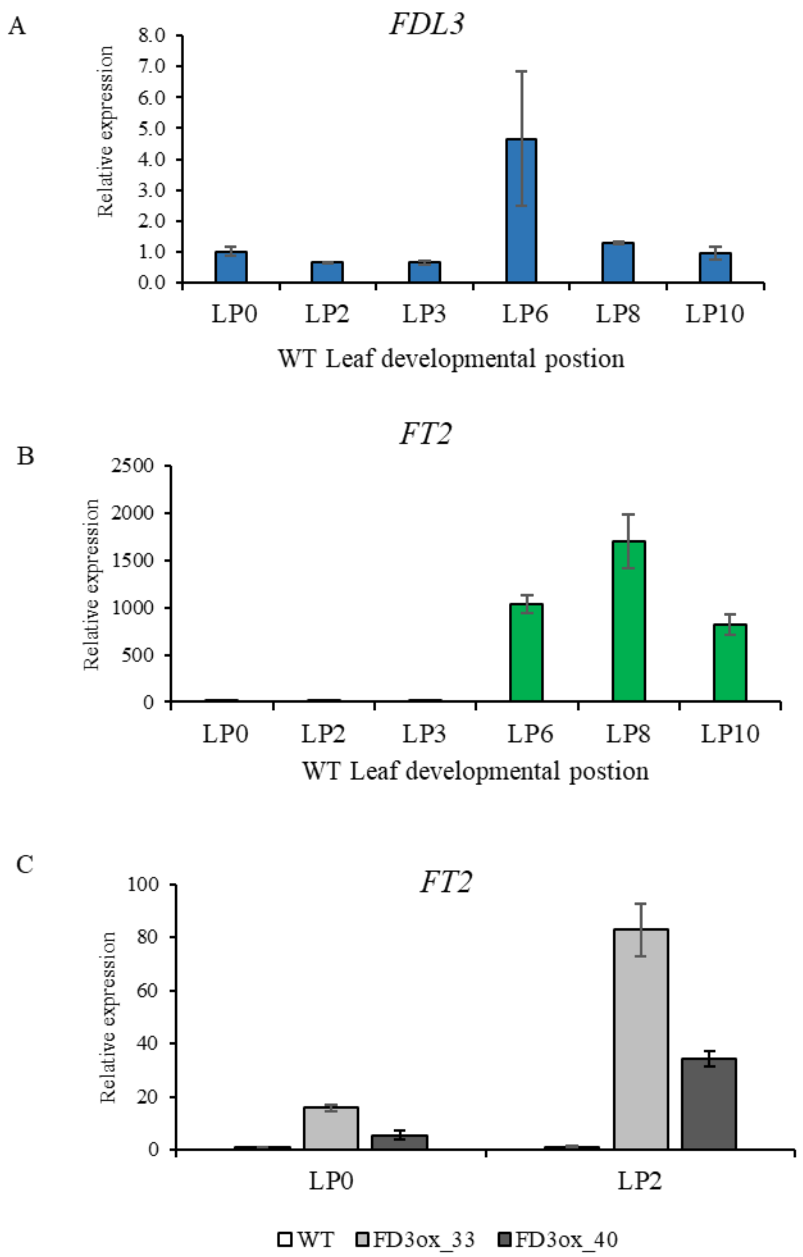

Supplementary Figure S9. *FDL3* and *FT2* expression in leaves at different developmental stages. (A-B) Plants were grown for 2 months in long days (16 hr light/8 hr dark). (C). Leaves were collected from WT and two independent events of *FDL3ox* plants grown for 2 months in long days (LDs), followed by 3 weeks in short days (SDs). (A-C) Leaves were collected at 2 hours after lights on. Leaf plastochron index 1 (LP1) was defined as the first leaf from the top, whose lamina length was equal to or larger than 1 cm.

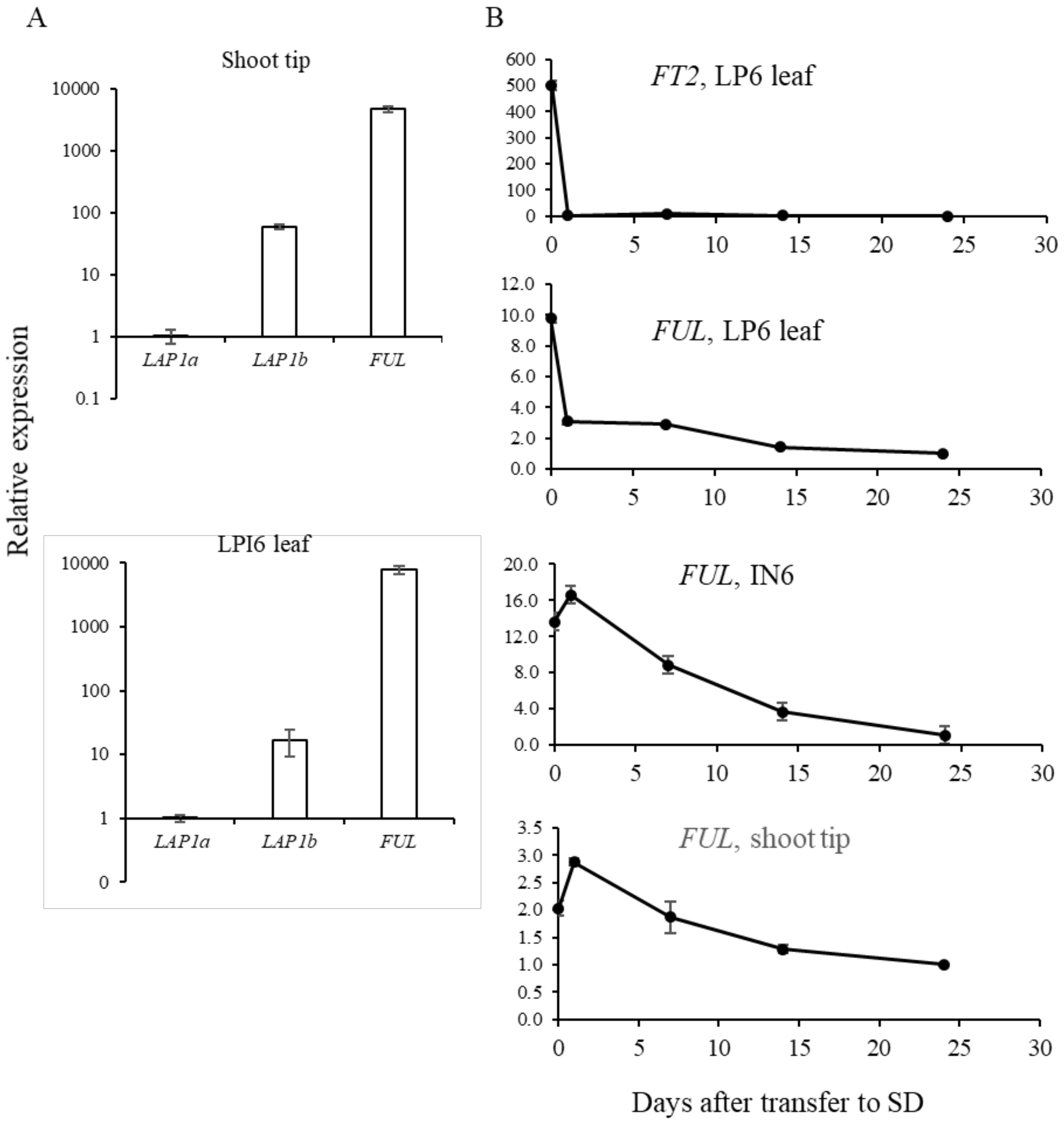

Supplementary Figure S10. Comparison of *LAP1a*, *LAP1b* and *FUL* expression levels in different tissues and time course of *FUL* downregulation in response to short days. (A) Comparative expression analysis of *LAP1a, LAP1b* and *FUL* in shoot apices, and LPI6 leaves. Tissues were collected from WT plants grown for 2 months in long daylengths. Expression was normalized against *UBQ2* and expressed as fold upregulation to the gene showing the lowest expression. (B) *FUL* expression was monitored in shoot apex, LPI6 leaf and IN6 after transfer to short daylengths. *FT2* expression in leaf is provided for comparison. All samples were collected 2 hours after the start of the daylight period.

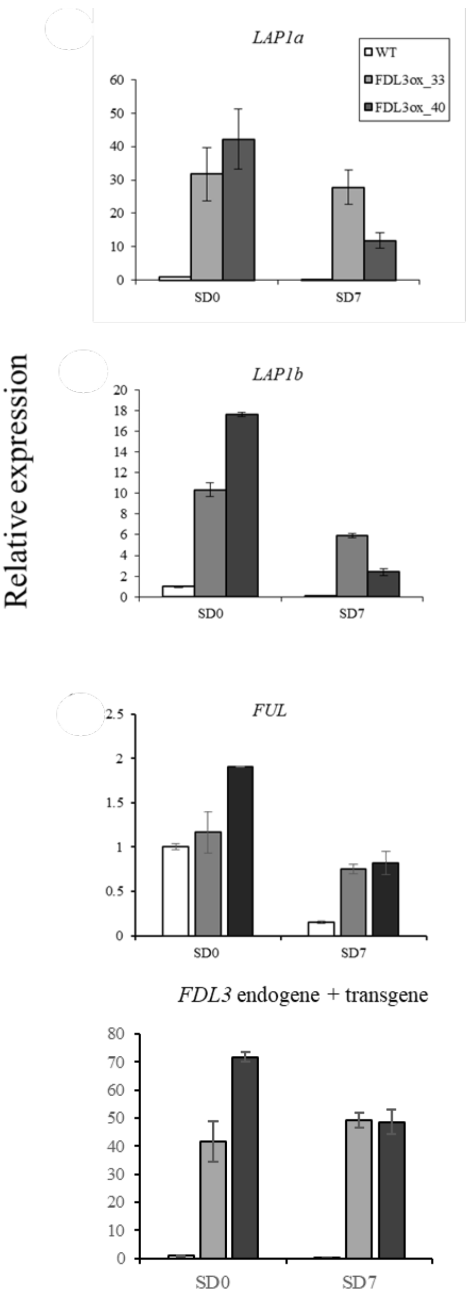

Supplementary Figure S11. *FDL3ox* and daylength alter *LAP1a*, *LAP1b* and *FUL* expression in shoot apices. WT and two independent events of *FDL3ox* (33 and 40) plants were grown for 2 months in long days (LDs). Shoot apices were collected in LDs (SD0) and after 7 days in SDs (SD7). Fold changes in transcript levels were relative to that of WT. The expression was normalized against *UBQ*. Samples were collected 2 hours after lights on.

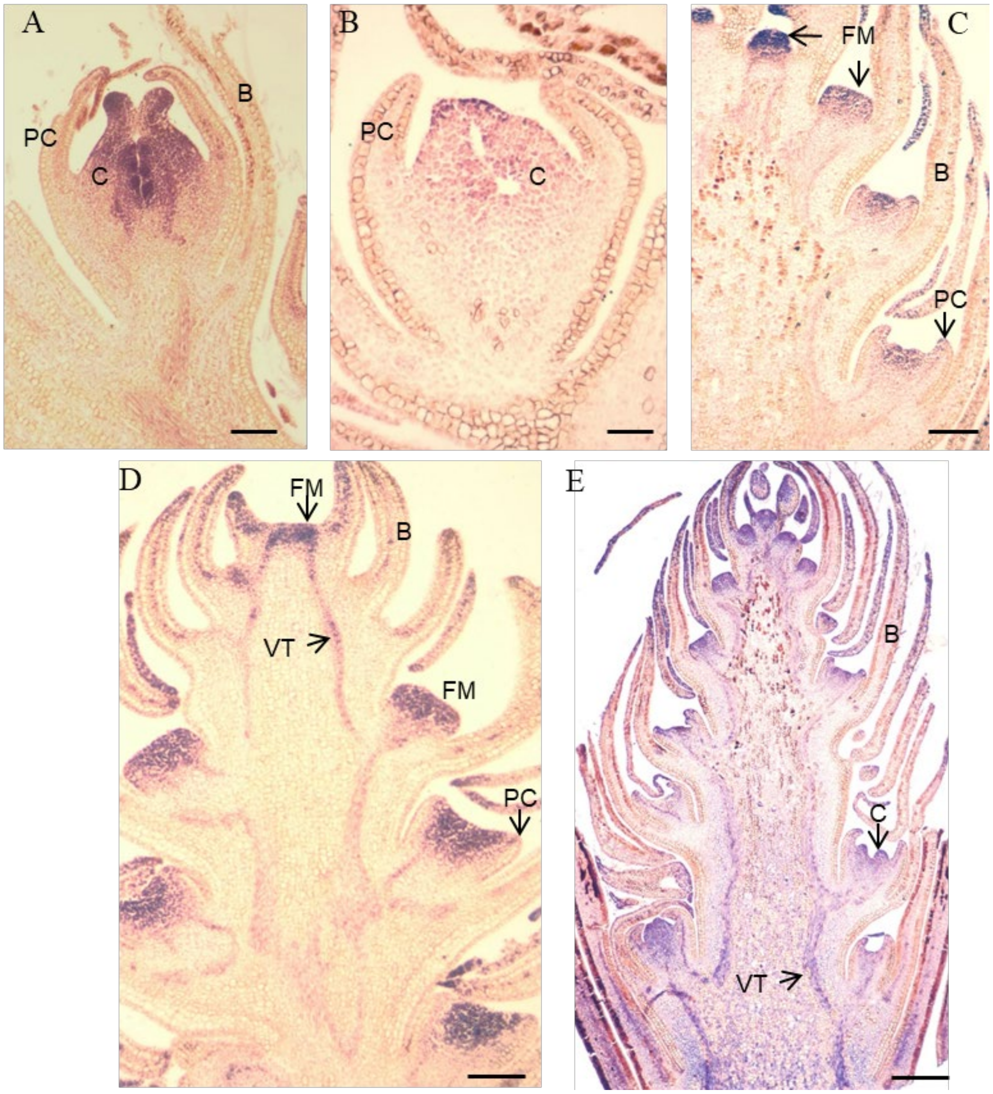

Supplementary Figure S12. *LAP1a* and *LAP1b* spatial expression in developing inflorescences. (A-E) *In situ* hybridizations of developing *P. trichocarpa* inflorescences. *LAP1b* expression in (A) differentiating female flower, (C) immature female catkin showing a gradient of early flower development stages, and (D) and immature male catkin (B) Negative control (*LAP1b* sense probe). (E) *LAP1a* expression in an immature female catkin. B, bract; C, carpel, FM, floral meristem; PC; perianth cup; VT, vascular trace. Scale bars = 100 µm (A, B, D) 200 µm (C), and 500 µm (E).

Supplementary Table S1. Primers used in this study

Primer	Sequence (5'-3')	Orientation	Purpose
FDL2.2F	ATGTGGTCATCGCCAGGAGC	Forward	FDL2.2 cloning
FDL2.2R	TCAAAATGGAGCTGTTGAGG	Reverse	
FDL3F	ATGTTGTCGCCAACAGATTG	Forward	FDL3 cloning
FDL3R	TCAAAATGGAGCTGTTGATG	Reverse	
FDL1F	ATGAGCACCAATAAAGTCTC	Forward	FDL1 With SRDX
FDL1R(SRDX)	TCAGGCGAATCCGAGTCTCAGTTCGAGATCCAGATCGAG AAATGGAGCCGTTGAGG	Reverse	
FDL2.1F	ATGTGGTCATCGCCAGGAGC	Forward	FDL2.1 With SRDX
FDL2.1R(SRDX)	TCAGGCGAATCCGAGTCTCAGTTCGAGATCCAGATCGAG AAATGGAGCTGTTGAGG	Reverse	
FDL2.2F	ATGTGGTCATCGCCAGGAGC	Forward	FDL2.2 With SRDX
FDL2.2R(SRDX)	TCAGGCGAATCCGAGTCTCAGTTCGAGATCCAGATCGAG AAATGGAGCTGTTGAGG	Reverse	
FDL3F	ATGTTGTCGCCAACAGATTG	Forward	FDL3 With SRDX
FDL3R(SRDX)	TCAGGCGAATCCGAGTCTCAGTTCGAGATCCAGATCGAG AAATGGAGCTGTTGATG	Reverse	
PdFDL1qF*	CAGCAACAACAACACCGACGACCATG	Forward	FDL1 qRT-PCR
PdFDL1qR	CAACTCGGTCGTGTAAGCCTGCTTCC	Reverse	
PdFDL2.1qF	CTAATTTTGCAGGCTTACACAGTTG	Forward	FDL2.1 qRT-PCR
PdFDL2.1qR	GAGCAAGTGGGATTAATGTATTCTC	Reverse	
PdFDL2.2qF	GGAAAGCAGTGATACTGTCCCTGTG	Forward	FDL2.2 qRT-PCR
PdFDL2.2qR	CTCAACTGTGTAAGCCTGCTTTCTAGC	Reverse	
PdFDL3qF	CCGATCAAGAACTTTCCATGACCCCA	Forward	FDL3 qRT-PCR
PdFDL3qR	CAGGCCTCTGAGGATGATCAGAGTTC	Reverse	
Pd18SrRNAqF	GGAATTGACGGAAGGGCACCACCAGGC	Forward	18SrRNA qRT-PCR
Pd18SrRNAqR	GGACATCTAAGGGCATCACAGACCTG	Reverse	
PdFULqF	CCGCTCCAATCCAACATCGAGC	Forward	FUL qRT-PCR
PdFULqR_	CAACATTTTCACTTCATCAGACTG	Reverse	
717FDL1qF	GAGAAGTAAGAAATCTATGCTTAGCCCAT	Forward	FDL1 qRT-PCR
717FDL1qR	CCTTTCTTTTCCCCTCCTTTCTTTTACAT	Reverse	
717FDL2.1qF	CAGGAATCTGGCTCTCCTTTTGAAAATT	Forward	FDL2.1 qRT-PCR
717FDL2.1qR	CAGCTTCACGCTCCAACTCAACT	Reverse	
717FDL2.2qF	GCTAGAAAGCAGGCTTACACAGTTGA	Forward	FDL2.2 qRT-PCR
717FDL2.2qR	GTGGGATTAATGTATTCTCAAAATGGAGC	Reverse	
717FDL3qF	TGGCCCTGGCTTTGATTTTCTTGAG	Forward	FDL3 qRT-PCR
717FDL3qR	AGATGAATCTAAACCCTCAAAAGGACTAG	Reverse	
717FT2qF	CTACCGGGGCGAACTTTGGGCAAGAGG	Forward	FT2 qRT-PCR
717FT2qR	TCATGGTCTCCTTCCACCGGAGCCAC	Reverse	
717UBQ2qF	TGTACTCTTTTGAAGTTGGTGT	Forward	UBQ qRT-PCR
717UBQ2qR	TCCAATGGAACGGCCATTAA	Reverse	
717LAP1aqF	GAGAAGGAGAAGAAGGATAAAGCTG	Forward	LAP1a qRT-PCR
717LAP1aqR	CCAAATATTCATGCTCCGTAACC	Reverse	
717LAP1bqF	AGATCAAGGAGAAGGAGAAAGCAC	Forward	LAP1b qRT-PCR
717LAP1bqR	ACCCAAATATTCATGTTCCAAAGC	Reverse	
717FULqF	CCACTCCAATCCAACATCGAG	Forward	FUL qRT-PCR
717FULqR	CAACATTTTCGCTTCATCAGACAG	Reverse	
717MADS28qF	GATGCTTCGCCATGTCAACG	Forward	MADS28 qRT-PCR
717MADS28qR	GAAAGTTACGAAATACATTCTTGTGCTC	Reverse	
717MADS14qF	GGATGCTTCGCCATGTCACTG	Forward	MADS14

717MADS14qR	TTATTGTGACTTAGCACTTGAAG	Reverse	qRT-PCR
717FUL-294	GCTCAAAGCTCGGGTAGATGTTC	Forward	3' RACE
717FUL-406	GCACTTAAGCACGTGAGATC	Forward	3' RACE nested
717MADS28-336	TGCAGGACAGGAATTGGATCC	Forward	3' RACE
717MADS28-403	GCTCTTAAGCGCATACGATC	Forward	3' RACE nested
717MADS14-297	GGCTAGGGTTGAACTCTTGCAG	Forward	3' RACE
717MADS14-370	GAACTTCAACACCTGGAGC	Forward	3' RACE nested
3' RACE adapter	GAGACTCGAGTCGACATCG	Reverse	3' RACE
NAC154q-F**	TCTTGCTGAGTACTATCACCCTTCTTC	Forward	NAC154
NAC154q-R	GCTACATGAGCCACCATATCATG	Reverse	qRT-PCR
GASA6_qF	TCAGCAAGTCATTTCCCTTTGTACC	Forward	GASA6 qRT-PCR
GASA6_qR	TGACGATGCCGGCCTCATAAAATTTC	Reverse	
4CL3-qF	GAGAGAAACGTTGCCAGGCATATAAC	Forward	4CL3 qRT-PCR
4CL3-qR	CCAAGCACGCTTGATTCAACACTAC	Reverse	
GA20ox5_F	CTAATTTAGCTAGGCTAGCAGCTATCTC	Forward	GA20ox5 qRT-PCR
GA20ox5_R	CTTTGAATGCGCTATAGGCAGAACAC	Reverse	
EXPA8_F	CACCCGCTGGGCCTTTATATATGCAGCAT	Forward	EXPA8 qRT-PCR
EXPA8_R	CCAACTGGGTTTCAGACCAAATTGGGGA	Reverse	

*Pd, *Populus deltoides*; 717, *P. tremula x P. alba* clone INRA717-IB. **Potri IDs: Potri.017G016700.1 (*NAC154*), Potri.001G254100.1 (*GASA6*), Potri.001G036900.1 (*4CL3*), Potri.019G057500.1 (*EXPA8*).

Supplementary Table S2. List of sequences used in phylogeny shown in Supplementary Figure S1A.

ID or accession no.	Name	Species	Database
Podel.02G017800.1	PdFDL1*	*Populus deltoides*	Phytozome: Populus deltoides WV94 v2.1
Podel.05G259400.1	PdFDL2.1*	*Populus deltoides*	
Podel.05G259400	PdFDL2.2*	*Populus deltoides*	
Podel.05G122500.1	PdFDL3*	*Populus deltoides*	
AT4G35900.1	FD	*Arabidopsis thaliana*	Phytozome: Arabidopsis thaliana TAIR10
AT2G17770.2	FDP	*Arabidopsis thaliana*	
GSVIVT01009970001	VvFDL1	*Vitis vinifera*	Phytozome: Vitis vinifera Genoscope.12X
GSVIVT01006332001	VvFDL2	*Vitis vinifera*	
AGK89941.1	AcFD	*Actinidia chinensis*	NCBI GenBank
PSS29994.1	AcFDL	*Actinidia chinensis*	
XP_027073256.1	CaFDL1	*Coffea arabica*	
XP_027077219.1	CaFDL2	*Coffea arabica*	
Solyc02g083520.2.1	SlFDL1	*Solanum lycopersicum*	
Solyc02g061990.2.1	SlFDL2	*Solanum lycopersicum*	
Aqcoe6G217300.1	AqcFDL	*Aquilegia coerulea*	Phytozome: Aquilegia coerulea v3.1
GSMUA Achr9T21040 001	MaFD1	*Musa Acuminata*	Phytozome: Musa acuminata v1

GSMUA Achr1T26400 001	MaFD2	*Musa Acuminata*	
GSMUA Achr5T11470 001	MaFD3	*Musa Acuminata*	
GSMUA Achr5T11220 001	MaFD4	*Musa Acuminata*	
GSMUA Achr5T17850 001	MaFD5	*Musa Acuminata*	
Os09g36910.1	OsFD1	*Oryza sativa*	Phytozome: Oryza sativa v7_JGI
Os06g50830.1	OsFD2	*Oryza sativa*	
Os02g58670.1	OsFD3	*Oryza sativa*	
Os08g43600.1	OsFD4	*Oryza sativa*	
Os06g50830.1	OsFD5	*Oryza sativa*	
Os06g50600.1	OsFD6	*Oryza sativa*	

*Protein sequence encoded by cDNAs reported in this paper were used rather than database predicted protein (models predict different splicing; see **Supplementary Figure** S1 for further details).

Supplementary Table S3. List of sequences used in phylogeny shown in Supplementary Figure S8A.

ID or accession no.	Name	Species	Database
Potri.010G154100.1	LAP1a	*Populus trichocarpa*	Phytozome: Populus trichocarpa v3.0
Potri.008G098500.1	LAP1b	*Populus trichocarpa*	
Potri.012G062300.1	PtrFUL	*Populus trichocarpa*	
Potri.017G099800.1	PtrMADS14	*Populus trichocarpa*	
Potri.004G115400.1	PtrMADS28	*Populus trichocarpa*	
AT1G69120.1	AP1	*Arabidopsis thaliana*	Phytozome: Arabidopsis thaliana TAIR10
AT1G26310.1	CAL	*Arabidopsis thaliana*	
AT5G60910.1	FUL	*Arabidopsis thaliana*	
AT3G30260.1	AGL79	*Arabidopsis thaliana*	
CAA67967.1	BpMADS3	*Betula pendula*	NCBI GenBank
CAA67969.1	BpMADS5	*Betula pendula*	
CAA67968.1	BpMADS4	*Betula pendula*	
GSVIVT01012250001	VvAP1	*Vitis vinifera*	Phytozome: Vitis vinifera Genoscope.12X
GSVIVT01008140001	VvFUL	*Vitis vinifera*	
GSVIVT01036549001	VvFUL-L	*Vitis vinifera*	
Solyc05g056620.1.1	MC	*Solanum lycopersicum*	Phytozome: Solanum lycopersicum iTAG2.4
Solyc03g114830.2.1	MBP7	*Solanum lycopersicum*	
Solyc06g069430.2.1	TM4	*Solanum lycopersicum*	
Solyc02g065730.1.1	MBP10	*Solanum lycopersicum*	
Solyc02g089210.2.1	MBP20	*Solanum lycopersicum*	

Supplementary Table S4. Relative expression levels (mean fold change ± SD) of *FDL* transcripts plotted in Figure 1.

	FDL1 (Mean fold change ± SD)[1]				
**Month**	**Leaf[2]**	**Reproductive bud[3]**	**Shoot apex[4]**	**Vegetative bud[5]**	**Shoot[6]**
**Sep**	96.8 ± 30.4	12.8 ± 4.4	17.0 ± 0	2.8 ± 0.3	20.6 ± 10.4
**Oct**	145.6 ± 30.9	48.0 ± 20.0	24.9 ± 0	6.3 ± 2.1	23.0 ± 6.2
**Nov**	187.3 ± 33.5	113.7 ± 30.3	44.3 ± 0	N/A	41.0 ± 19.2
**Dec**	291.9 ± 47.7	167.3 ± 74.3	49.2 ± 0	N/A	87.1 ± 59.3
**Jan**	187.2 ± 27.5	145.6 ± 60.0	29.8 ± 0	7.9 ± 2.1	29.5 ± 18.0
**Feb**	44.9 ± 6.1	117.2 ± 32.3	46.9 ± 0	7.6 ± 2.9	17.4 ± 9.1
**Mar**	2.7 ± 0.4	1.0 ± 0.3	1.3 ± 0	N/A	4.8 ± 4.8
**Apr**	1.1 ± 0.4	4.5 ± 0.8	1.0 ± 0	4.2 ± 6.7	1.1 ± 0.6
**May**	12.4 ± 2.0	13.6 ± 6.1	1.7 ± 0	2.4 ± 3.3	3.0 ± 2.1
**Jun**	43.8 ± 16.6	28.1 ± 11.8	2.4 ± 0	2.4 ± 0.7	8.5 ± 3.3
**Jul**	18.2 ± 8.3	20.1 ± 7.1	2.4 ± 0	1.0 ± 0.5	13.9 ± 4.6
**Aug**	17.5 ± 2.3	29.9 ± 15.0	5.5 ± 0	1.9 ± 0.8	27.9 ± 7.9
	FDL2.1 (Mean fold change ± SD) [1]				
**Month**	**Leaf[2]**	**Reproductive bud[3]**	**Shoot apex[4]**	**Vegetative bud[5]**	**Shoot[6]**
**Sep**	199.5 ± 195.2	95.4 ± 34.7	33.9 ± 0	18.0 ± 12.0	34.9 ± 6.6
**Oct**	84.2 ± 89.2	46.7 ± 9.6	12.7 ± 0	7.5 ± 5.3	55.1 ± 45.4
**Nov**	11.2 ± 13.1	29.8 ± 9.6	1.2 ± 0	N/A	23.6 ± 22.9
**Dec**	17.9 ± 17.9	12.4 ± 3.2	1.0 ± 0	N/A	12.5 ± 15.2
**Jan**	6.2 ± 6.6	19.3 ± 7.6	1.0 ± 0	1.8 ± 1.5	1.5 ± 0.9
**Feb**	5.8 ± 6.9	1.2 ± 0.7	1.9 ± 0	1.9 ± 1.5	1.9 ± 2.5
**Mar**	45.3 ± 50.7	8.0 ± 2.0	8.4 ± 0	N/A	8.2 ± 4.6
**Apr**	1.9 ± 2.5	3841.3 ± 1178.1	25.3 ± 0	79.4 ± 50.2	50.7 ± 39.0
**May**	9.8 ± 13.5	1475.3 ± 178.2	18.4 ± 0	68.2 ± 51.7	67.9 ± 60.8
**Jun**	8.0 ± 10.3	895.2 ± 81.7	23.3 ± 0	54.4 ± 38.1	32.2 ± 16.5
**Jul**	6.0 ± 7.6	484.4 ± 83.4	29.2 ± 0	36.4 ± 33.0	67.8 ± 34.4
**Aug**	10.1 ± 11.1	265.1 ± 29.4	30.6 ± 0	24.2 ± 17.8	49.3 ± 48.4
	FDL2.2 (Mean fold change ± SD)[1]				
**Month**	**Leaf[2]**	**Reproductive bud[3]**	**Shoot apex[4]**	**Vegetative bud[5]**	**Shoot[6]**
**Sep**	191.1 ± 123.1	230.5 ± 145.1	197.6 ± 0	42.5 ± 27.4	191.1 ± 123.1
**Oct**	163.6 ± 64.8	119.8 ± 99.9	46.7 ± 0	11.4 ± 6.8	163.6 ± 64.8
**Nov**	54.6 ± 26.9	34.5 ± 32.2	2.1 ± 0	N/A	54.6 ± 26.9
**Dec**	14.0 ± 7.2	8.6 ± 9.2	1.1 ± 0	N/A	14.0 ± 7.2
**Jan**	1.1 ± 0.4	17.9 ± 16.8	1.0 ± 0	1.0 ± 0.3	1.1 ± 0.4
**Feb**	1.3 ± 0.4	1.3 ± 1.2	2.7 ± 0	1.2 ± 0.4	1.3 ± 0.4
**Mar**	31.0 ± 2.9	15.5 ± 7.2	57.1 ± 0	N/A	31.0 ± 2.9
**Apr**	211.5 ± 115.0	19149.7 ± 19680.5	168.9 ± 0	155.4 ± 72.1	211.5 ± 115.0

**May**	304.9 ± 81.1	6980.5 ± 6003.7	130.1 ± 0	133.7 ± 38.8	304.9 ± 81.1
**Jun**	88.3 ± 8.6	4095.8 ± 3326.6	178.1 ± 0	104.9 ± 29.9	88.3 ± 8.6
**Jul**	364.7 ± 251.5	2072.7 ± 1830.9	228.1 ± 0	63.6 ± 13.6	364.7 ± 251.5
**Aug**	179.3 ± 54.7	787.3 ± 476.6	161.3 ± 0	45.5 ± 16.4	179.3 ± 54.7
	FDL3 **(Mean fold change ± SD)** [1]				
**Month**	**Leaf**[2]	**Reproductive bud**[3]	**Shoot apex**[4]	**Vegetative bud**[5]	**Shoot**[6]
**Sep**	192.0 ± 26.6	10.2 ± 7.1	26.5 ± 0	2.9 ± 0.8	1.7 ± 0.4
**Oct**	78.4 ± 7.8	7.6 ± 1.5	7.9 ± 0	1.0 ± 0.2	3.0 ± 0.7
**Nov**	16.4 ± 5.4	2.8 ± 0.8	1.0 ± 0	N/A	1.3 ± 0.2
**Dec**	30.3 ± 5.5	1.2 ± 0.8	1.0 ± 0	N/A	6.9 ± 1.0
**Jan**	10.9 ± 2.7	2.2 ± 0.6	1.7 ± 0	1.0 ± 0.3	1.5 ± 1.0
**Feb**	6.0 ± 2.8	1.5 ± 0.4	6.6 ± 0	1.4 ± 0.5	1.0 ± 0.4
**Mar**	45.5 ± 13.6	2.8 ± 1.5	25.8 ± 0	N/A	1.7 ± 0.9
**Apr**	9.4 ± 0.9	13.1 ± 6.3	14.8 ± 0	5.2 ± 0.5	3.5 ± 0.04
**May**	1.1 ± 0.4	17.0 ± 2.2	27.1 ± 0	4.1 ± 1.1	3.1 ± 1.3
**Jun**	7.8 ± 4.1	19.4 ± 2.7	36.3 ± 0	4.2 ± 0.3	4.8 ± 0.5
**Jul**	4.5 ± 1.4	14.6 ± 3.9	24.3 ± 0	2.8 ± 0.6	5.8 ± 1.8
**Aug**	7.5 ± 2.6	12.2 ± 3.5	41.2 ± 0	3.0 ± 0.7	3.6 ± 0.9

[1]n=3 biological replicates for all collected tissue types, except shoot apex sample (n=1). Shoot apices collected from the 3 branches were pooled together because of limited amount of tissue.

[2]Leaf: preformed leaves in terminal buds were collected from September to March before terminal bud flush. Fully expanded leaves at node #9, #10, #11 from the base of shoot (floral zone) were collected from April to August after terminal bud flush.

[3]Reproductive bud: axillary reproductive buds were collected after removing bud scales.

[4]Shoot apex: bud scales and preformed leaves were removed from terminal buds

[5]Vegetative bud: axillary vegetative buds were collected after removing bud scales.

[6]Shoot: preformed shoots in terminal buds were collected from September to March. Two to 3 cm-long shoot section were collected from the node #9 to #11 zone (from the base of shoot) from April to August.

Supplementary Table S5. Relative expression levels (mean fold change ± SD) of *FUL* transcripts plotted in Figure 8E.

	***FUL* (Mean fold change ± SD)**				
**Month**	**Leaf**	**Reproductive bud**	***Shoot apex**	**Vegetative bud**	**Shoot**
**Sep**	1.1±0.3	2.3±0.2	2.5±0.1	1.5±0.3	2.5±0.2
**Oct**	1.1±0.4	4.4±0.3	6.9±0.9	5.2±0.7	4.9±0.7
**Nov**	3.9±1.9	14.7±0.8	22.1±2.5	N/A	7.8±1.8
**Dec**	5.9±2.8	6.3±0.5	5.7±0.2	N/A	19.3±2.9
**Jan**	10.6±1.7	33.5±6.5	144.2±21.4	67.6±6	26.4±2.1
**Feb**	9.2±1.8	16.1±2.9	80.3±18.3	20.8±2.7	56.8±13.5
**Mar**	13.9±2.5	4.3±0.8	51.2±5.4	N/A	26±6.6
**Apr**	23.1±7	1.9±0.5	27.8±6.2	2.3±0.6	11.4±3.7
**May**	31.5±6.6	1.9±0.5	13.8±3.4	2.1±0.2	7.3±2.6
**Jun**	22±1.6	1.3±0.1	4.8±3	1±0.1	4.5±2.8
**Jul**	19.8±3.6	1±0.1	3.4±1.7	1.2±0	2.9±1.2
**Aug**	16.6±2.4	1±0.1	1.1±0.6	1±0.1	1.4±1.4

*For the shoot apex there was only one biological replicate and ±SD is from three technical replicates.
